# Gemcitabine Modulates HLA-I Regulation to Improve Tumor Antigen Presentation by Pancreatic Cancer Cells

**DOI:** 10.3390/ijms25063211

**Published:** 2024-03-11

**Authors:** Alaina C. Larson, Shelby M. Knoche, Gabrielle L. Brumfield, Kenadie R. Doty, Benjamin D. Gephart, Promise R. Moore-Saufley, Joyce C. Solheim

**Affiliations:** 1 Eppley Institute for Research in Cancer & Allied Diseases, Fred & Pamela Buffett Cancer Center, University of Nebraska Medical Center, Omaha, NE 68198, USA; 2 Departments of Psychology and Biology, University of Nebraska Omaha, Omaha, NE 68192, USA

**Keywords:** gemcitabine, human leukocyte antigen class I, immunoproteasome, pancreatic cancer, immunomodulatory, chemotherapy, peptide, antigen presentation, neoantigen

## Abstract

Pancreatic cancer is a lethal disease, harboring a five-year overall survival rate of only 13%. Current treatment approaches thus require modulation, with attention shifting towards liberating the stalled efficacy of immunotherapies. Select chemotherapy drugs which possess inherent immune-modifying behaviors could revitalize immune activity against pancreatic tumors and potentiate immunotherapeutic success. In this study, we characterized the influence of gemcitabine, a chemotherapy drug approved for the treatment of pancreatic cancer, on tumor antigen presentation by human leukocyte antigen class I (HLA-I). Gemcitabine increased pancreatic cancer cells’ HLA-I mRNA transcripts, total protein, surface expression, and surface stability. Temperature-dependent assay results indicated that the increased HLA-I stability may be due to reduced binding of low affinity peptides. Mass spectrometry analysis confirmed changes in the HLA-I-presented peptide pool post-treatment, and computational predictions suggested improved affinity and immunogenicity of peptides displayed solely by gemcitabine-treated cells. Most of the gemcitabine-exclusive peptides were derived from unique source proteins, with a notable overrepresentation of translation-related proteins. Gemcitabine also increased expression of select immunoproteasome subunits, providing a plausible mechanism for its modulation of the HLA-I-bound peptidome. Our work supports continued investigation of immunotherapies, including peptide-based vaccines, to be used with gemcitabine as new combination treatment modalities for pancreatic cancer.

## 1. Introduction

Pancreatic cancer is a fatal disease with a bleak overall five-year survival rate of 13% [1]. Because only a minor population of pancreatic cancer patients present with resectable disease, the majority of individuals receive systemic chemotherapy as their sole means of disease management [2]. Gemcitabine/nab-paclitaxel and FOLFIRINOX (folinic acid, 5-fluorouracil, irinotecan, and oxaliplatin) are the two standardized chemotherapeutic regimens approved for the treatment of pancreatic cancer [3]. Although both regimens extend survival, the latter has a higher incidence of adverse effects, and disease progression ultimately ensues for most patients on either regimen [4]. Consequently, there is a critical need to identify additional therapeutic options for patients, with interest shifting towards immunotherapies and potential combinatory routes.

Historically, immunotherapies, particularly single-agent immune checkpoint inhibitors, have held little success in the treatment of pancreatic cancer, and their inadequacy is thought to be a byproduct of the immunosuppressive microenvironment characteristic of this disease [5,6,7,8]. In the subsets of pancreatic cancer patients where immuno-promotive events exist (e.g., enriched CD8^+^ T cell populations), survival outcomes are improved [9,10,11,12]. It has thus become of interest to pinpoint therapeutic mechanisms to overcome innate suppression and revitalize anti-tumor responses against pancreatic cancer.

Studies have implicated select chemotherapy drugs, including gemcitabine, as potent modifiers of the immune system [13]. To date, gemcitabine has demonstrated a propensity to influence the behaviors and relative abundance of several immune cell populations, including reduction of circulating immunosuppressive cells [14], enhancement of NK cell infiltration and NK cell-mediated cytotoxicity [15,16], and increased cross-priming of tumor-specific cytotoxic T cells [17]. Previous findings showed that gemcitabine upregulated expression of the immunostimulatory complex human leukocyte antigen class I (HLA-I) and its murine form at the protein level [18]. However, robust analysis concerning gemcitabine’s regulation of HLA-I expressed by pancreatic cancer cells has never been reported, and information beyond protein expression is critical for determining the clinical appreciation of this finding.

HLA-I molecules present intracellularly derived peptides to cytotoxic T cells and thus are responsible for instigating T cell-mediated lysis of malignant cells [19]. HLA-I is a heterodimer complex, composed of the beta-2-microglobulin (β_2_m) light chain and one of three heavy chains (HLA-A, HLA-B, or HLA-C) [19]. The polymorphic nature of the heavy chain genes results in many allelic forms of HLA-I which are consequently referred to as allotypes (e.g., HLA-A2, HLA-A24) [19,20]. Following assembly, HLA-I molecules are loaded in the endoplasmic reticulum (ER) with peptides generated by the proteasome [19]. Though multiple proteasomes exist, the three catalytic subunits (β1i, β2i, β5i) of the interferon (IFN)/tumor necrosis factor alpha (TNFα)-inducible immunoproteasome have been found to generate higher quality peptides (e.g., higher affinity) for HLA-I binding [21]. In conjunction, several groups have reported stronger cytotoxic responses against immunoproteasome-created epitopes [22,23,24], and a recent in vitro study noted that overexpression of select immunosubunits generated a more immunogenic peptide repertoire capable of inducing T cell-mediated reactivity against melanoma cells [24].

Findings relating to the association of HLA-I expression and patient outcome vary in pancreatic cancer [25,26,27,28]. Still, HLA-I molecules must be present within the tumor microenvironment for cytotoxic T cell-mediated responses to occur, and high lymphocyte infiltration is considered a favorable prognostic factor in pancreatic cancer [29,30]. The presence of high quality tumor-specific antigens (which would be displayed by HLA-I molecules) were also positively correlated with long-term pancreatic cancer patient survival [31]. Thus, therapeutic interventions to stimulate HLA-I surface expression and facilitate its binding of high quality peptides could be promising investigational approaches for use in combination treatments that promote T cell activity.

With the knowledge that gemcitabine enhanced HLA-I protein expression in models of pancreatic cancer and that proteasome composition can potentiate T cell anti-tumor intervention [18,24], we sought to identify gemcitabine’s influence on antigen presentation by pancreatic cancer cells. In this study, we evaluated gemcitabine’s regulation of HLA-I at multiple levels. In conjunction, we showed that the expression of select immunoproteasome subunits was instigated by gemcitabine in pancreatic cancer cells, and our mass spectrometry analysis revealed that gemcitabine-treated cells presented a subset of unique, higher quality HLA-I-bound peptides.

## 2. Results

### 2.1. Gemcitabine Increases HLA-I Expression by Pancreatic Cancer Cells

**Figure 1:**
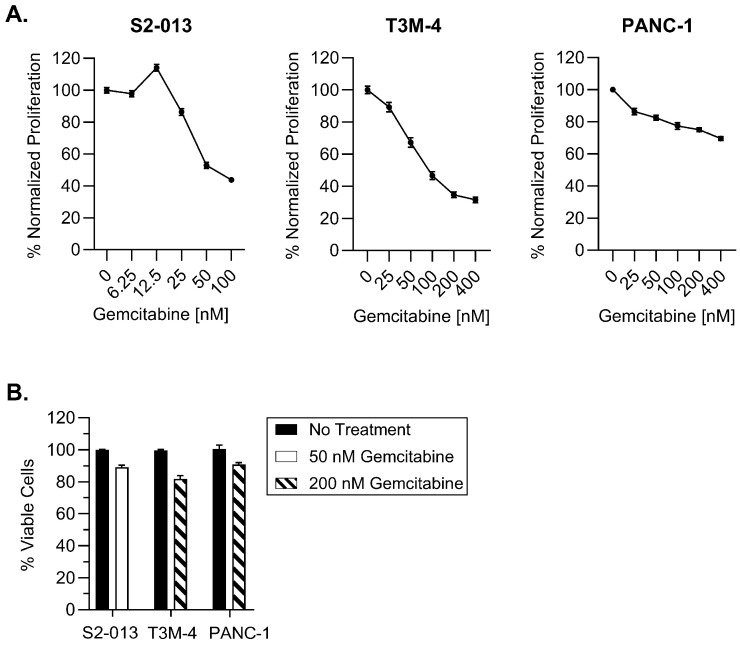
Selected gemcitabine concentrations maintain viability of pancreatic cancer cells. Cells were left untreated or exposed to gemcitabine for 72 h. (**A**) Proliferation was monitored with MTT assays. (**B**) Cytotoxicity of optimized gemcitabine concentrations were evaluated using trypan blue exclusion. Each error bar represents the standard error of the mean from at least three biological replicates (GraphPad PRISM v.10.2.0). Data were normalized to the untreated control for each cell line (**A**,**B**).

In order to determine gemcitabine’s effects on antigen presentation, we began first by assessing its influence on cell proliferation. Three human pancreatic cancer cell lines (S2-013, T3M-4, and PANC-1) were treated with varying concentrations of gemcitabine for 72 h. MTT assays demonstrated proliferative reduction in a concentration-dependent manner, particularly for the S2-013 and T3M-4 cells. PANC-1 growth was impacted to a lesser degree and reflects findings from previous studies [32,33] (Figure 1A).

Because our ultimate intention is to assess the impact of gemcitabine on improving HLA-I display of tumor antigens, we found it important to select concentrations which were not excessively toxic to the pancreatic cancer cell lines. We contend that lower concentrations would better discern the ability of gemcitabine to serve as a priming mechanism and render pancreatic tumor cells susceptible for T cell-mediated destruction. In line with this objective, our optimized doses had only a mild impact on viability of the S2-013, T3M-4, and PANC-1 cells following a 72-h gemcitabine incubation (Figure 1B).

In an effort to monitor gemcitabine’s influence on tumor antigen presentation, we started by investigating its effects on regulation of HLA-I expression. In its peptide-free state, HLA-I exists as a heterodimer composed of a heavy chain and light chain. The HLA-I type is determined by the particular heavy chain (i.e., HLA-A, HLA-B, or HLA-C) that associates with the conserved light chain (β_2_m) [19]. We assessed gemcitabine’s impact on surface HLA-I allotype expression in addition to monitoring expression of individual constituents of the HLA-I molecule at the RNA and protein levels.

**Figure 2:**
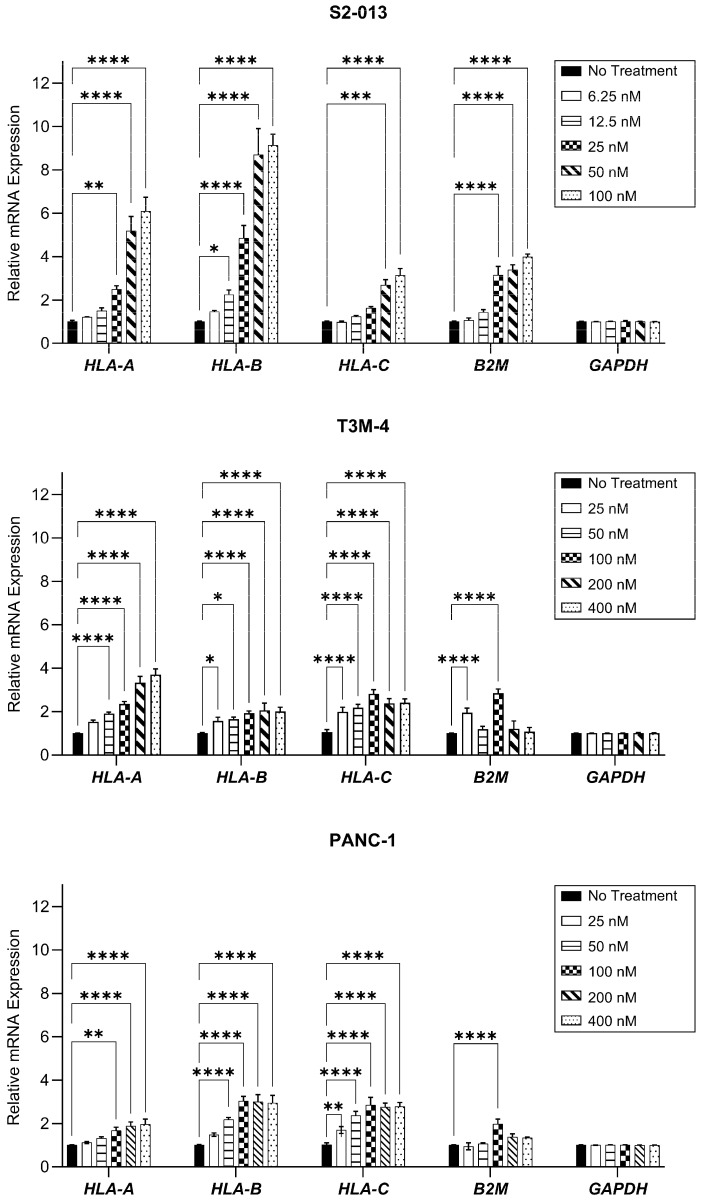
HLA-I mRNA transcripts are enhanced by gemcitabine. Pancreatic cancer cell lines were treated with gemcitabine at varying concentrations for 48 h. mRNA levels were assessed via qRT-PCR, with primers specific for *HLA-A*, *HLA-B*, *HLA-C*, or *B2M* genes. Each error bar represents the standard error of the mean from at least three biological replicates. Data were normalized to the reference gene (*GAPDH*) and fold change was compared to the untreated control. Statistical significance was analyzed using two-way ANOVA with Bonferroni’s post-test correction for multiple comparisons (GraphPad PRISM, v.10.2.0). The asterisks indicate the following *p*-values: * *p* < 0.05, ** *p* < 0.01, *** *p* < 0.001, **** *p* < 0.0001.

HLA-I mRNA levels were evaluated after a 48-h treatment period. Gemcitabine increased transcripts of the heavy chains *HLA-A*, *HLA-B*, and *HLA-C*, as well as the light chain, *B2M*, in all three cell lines, though the magnitude of this increase differed among treatment concentrations and cell types (Figure 2). In the evaluated cell lines, optimized concentrations of gemcitabine (which were ultimately used for monitoring surface HLA-I) conferred increases in transcripts of all heavy chains (Figure 2). However, *B2M* mRNA was only significantly enhanced in the S2-013 cells at this concentration (Figure 2).

**Figure 3:**
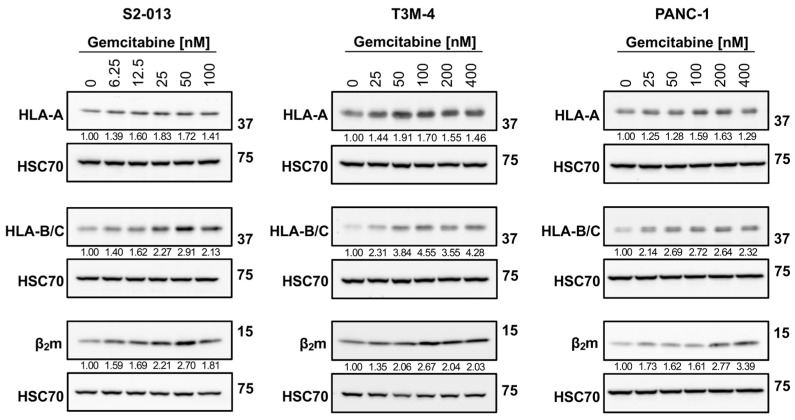
Gemcitabine augments protein expression of HLA-I. S2-013, T3M-4, and PANC-1 cells were treated with various concentrations of gemcitabine for 72 h. Western blot analysis monitored changes in total protein levels of HLA-I components. Antibodies were specific for the HLA-A heavy chain (EP1395Y), HLA-B and HLA-C heavy chains (HC10), or the β_2_m light chain (EP2978Y). HSC70 was used as a loading control. Blots are representative of at least three biological replicates. Numbers below bands indicate densitometric quantification performed with ImageLab (v.6.1). Protein expression is relative first to the respective loading control and then normalized to the untreated sample.

As shown with western blotting, gemcitabine had a mild impact on HLA-A protein expression in S2-013 and PANC-1 cells, but HLA-B/C proteins were strongly increased with treatment (Figure 3). (Antibodies specific for these individual HLA-B and HLA-C heavy chains were unavailable, so an antibody recognizing both HLA-B and HLA-C was used). In the T3M-4 cells, the degree of stimulation for all heavy chains was largest, and preferential augmentation of HLA-B/C over HLA-A was observed after treatment (Figure 3). Gemcitabine enhanced expression of the light chain, β_2_m, in all three cell lines (Figure 3).

**Figure 4:**
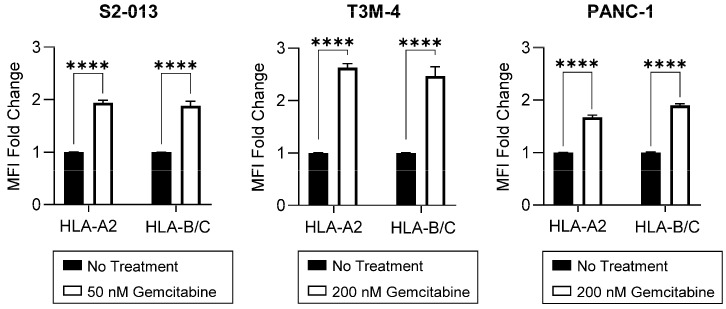
HLA-I surface expression is stimulated by gemcitabine. Cells were left untreated or treated with gemcitabine for 72 h. Surface expression of HLA-A2 (mAb BB7.2) or HLA-B/C (mAb HLA-B/C) was measured using flow cytometry. Each error bar represents the standard error of the mean from at least three biological replicates. MFI fold change represents median florescent intensity of the experimental group compared to the control group. Statistical significance was analyzed using an unpaired *t*-test (GraphPad Prism, v.10.2.0). The asterisks indicate the following *p*-values: **** *p* < 0.0001.

We next evaluated the impact of gemcitabine on surface HLA-I expression, a critical level of regulation since membrane-resident HLA-I molecules engage cytotoxic T cells. Expression of surface HLA-A2, a globally prevalent allotype [34], was increased by treatment in the S2-013, T3M-4, and PANC-1 cells (Figure 4). In tandem, gemcitabine also augmented surface HLA-B/C on all cell lines (Figure 4). Despite the apparent biased stimulation of HLA-B/C at the total protein level, gemcitabine-induced amplification of surface HLA-B/C molecules was similar to that of HLA-A2 in most cell lines (Figure 4). Competition between heavy chains for association with β_2_m [35], availability of suitable peptide ligands for binding [36], and/or variations in surface retention may explain this observation [37].

### 2.2. Gemcitabine Enhances Surface Stability of HLA-I on Pancreatic Cancer Cells

**Figure 5:**
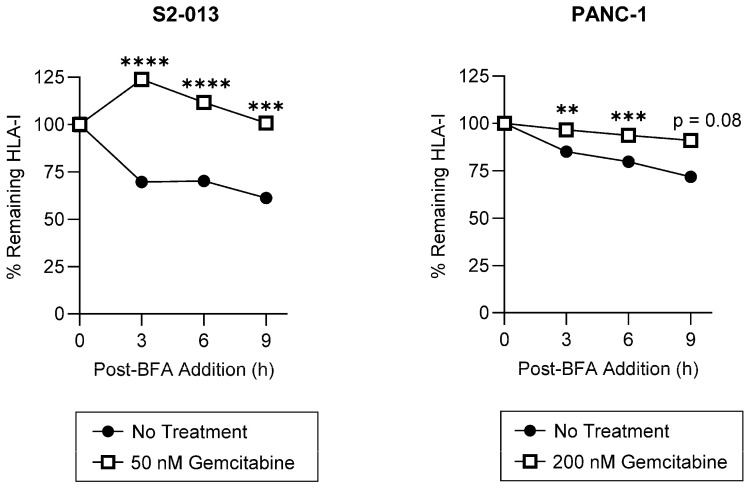
HLA-I surface stability is increased by gemcitabine. S2-013 and PANC-1 cells were left untreated or treated with gemcitabine for 72 h. Cells were incubated in the presence of Brefeldin A (BFA) to prevent additional trafficking of HLA-I molecules to the surface. Surface retention of HLA-I was monitored with flow cytometry with the pan-reactive mAb W6/32. Each point represents normalization to the 0-h time point. For each cell line, a representative stability plot depicts findings from at least three biological replicates with error bars indicating the standard error of the mean. Statistical significance was analyzed using an unpaired *t*-test (GraphPad PRISM, v.10.2.0). The asterisks indicate the following *p*-values: ** *p* < 0.01, *** *p* < 0.001, **** *p* < 0.0001.

Surface retention of HLA-I molecules is a critical mediator of T cell recognition. To assess gemcitabine’s impact on HLA-I surface stability, we used a brefeldin A (BFA) assay. BFA disrupts trafficking between the ER and the Golgi apparatus and thus prevents newly synthesized HLA-I molecules from reaching the surface [38]. HLA-I surface retention following addition of BFA to the cell media was monitored using W6/32, a monoclonal antibody recognizing peptide-bound HLA-A, HLA-B, and HLA-C complexes [39,40]. Gemcitabine increased HLA-I surface stability on the S2-013 cells, nearly doubling the proportion of membrane-resident HLA-I complexes retained at the evaluated time points (Figure 5). HLA-I surface stability was also significantly enhanced following treatment of the PANC-1 cells, albeit to a lesser degree (Figure 5). Such results suggest gemcitabine treatment may increase presentation of strongly bound peptides since, in the absence of peptide ligands, surface HLA-I molecules are denatured and less stable [41,42].

**Figure 6:**
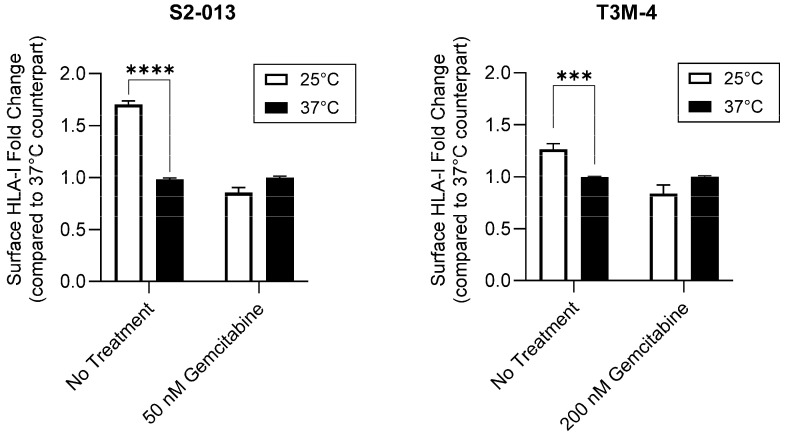
Gemcitabine mitigates HLA-I-binding of low affinity peptides. Pancreatic cancer cells were untreated or treated with gemcitabine at indicated concentrations. Respective plates were stored at 25 °C for at least 4 h prior to the conclusion of the 72-h treatment incubation. Surface expression of HLA-I (mAb W6/32) was measured via flow cytometry. Each error bar represents the standard error of the mean from three biological replicates. Data were normalized to their respective 37 °C counterpart. Statistical significance was analyzed using an unpaired *t*-test (GraphPad PRISM, v.10.2.0). The asterisks indicate the following *p*-values: *** *p* < 0.001, **** *p* < 0.0001.

Peptide composition and affinity for a particular HLA-I allotype is a major determinant of the HLA-I complex’s surface stability [43,44]. Thus, we sought to determine if gemcitabine’s impact on surface retention was in part derived from its favorable modulation of the HLA-I-bound peptidome. To provide evidence that gemcitabine was altering peptide ligand presentation by HLA-I molecules, we utilized a temperature-dependent assay in which gemcitabine-treated pancreatic cancer cells were exposed to physiological (37 °C) or low (25 °C) temperatures. Peptide loading by HLA-I molecules is a temperature-regulated process, and it has been previously shown that surface expression of HLA-I is increased at low temperatures [41,45]. Reduced dissociation of the β_2_m light chain as well as increased flexibility in the HLA-I peptide binding groove have been observed at low temperatures [45,46]. Such changes are believed to enable HLA-I surface presentation of suboptimal peptides, which are normally inadmissible for display at physiological temperatures [45,46]. In the evaluated pancreatic cancer cells, HLA-I surface expression was not increased at 25 °C post-gemcitabine treatment, indicating a lack of low affinity peptide binding and presentation by HLA-I molecules (Figure 6). However, in the absence of treatment, the characteristic stimulation of HLA-I surface expression was observed at the tested low temperature (Figure 6). These results thus support that gemcitabine reduces the relative availability of low affinity peptides for HLA-I binding and prompted a more robust investigation of gemcitabine’s impact on the immunopeptidome.

### 2.3. Gemcitabine Induces Presentation of Unique, Higher Quality Peptides by S2-013 Cells

**Figure 7:**
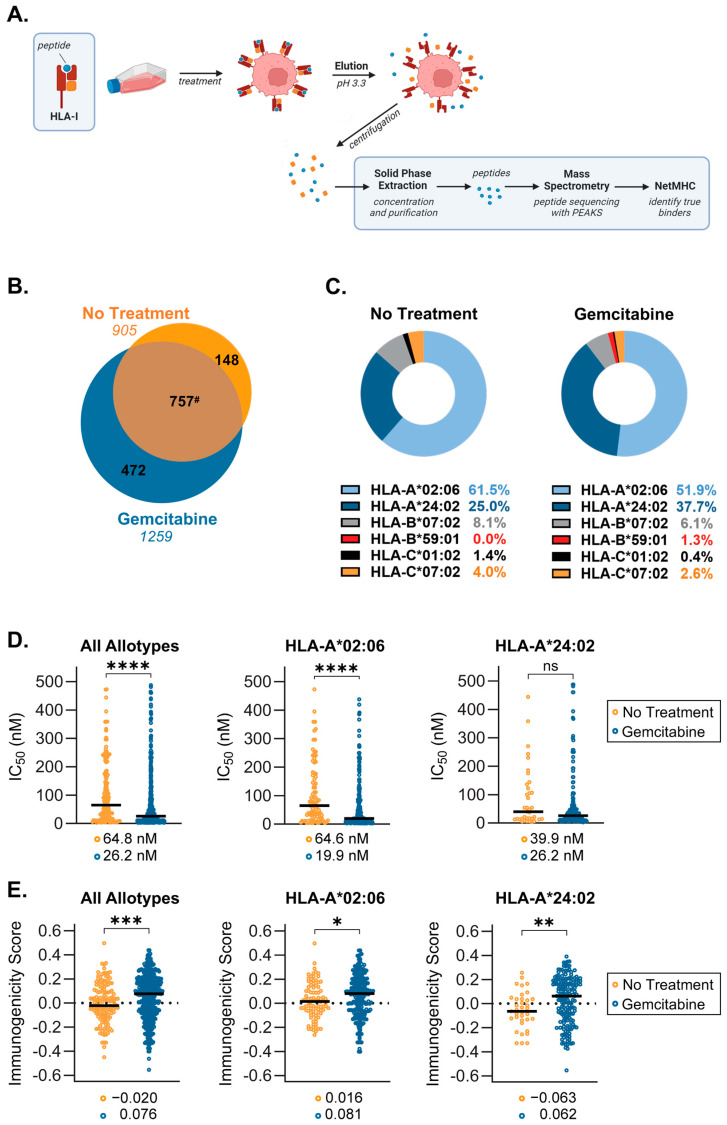
Gemcitabine induced alterations in the immunopeptidome of S2-013 pancreatic cancer cells. S2-013 cells were incubated in the absence or presence of gemcitabine (50 nM) for 72 h. HLA-I-bound peptides were collected via mild acid elution and analyzed using mass spectrometry. Peptide data from three separate experiments were pooled and reported as such. All panels represent peptides which were assigned as putative binders (NetMHC IC_50_ of ≤500 nM) to at least one of the HLA-I allotypes expressed by the cell line. (**A**) Schematic depicting mild acid elution (MAE) of HLA-I-presented peptides. (**B**) Venn diagram displaying peptides exclusive to the no treatment (orange) and gemcitabine-treated (blue) cohorts. Peptide ligands which were shared among groups (brown) are denoted in the overlapping region. The numbers in italics indicate total peptide numbers. (**C**) Donut charts showing the distribution of peptide binding between HLA-I allotypes. (**D**) NetMHC-predicted binding affinity between peptide/HLA-I complexes, wherein stronger binders are indicated by lower IC_50_ values. (**E**) IEDB-predicted immunogenicity scores. More positive scores suggest more immunogenic peptides. Black bars denote median scores, which are also listed directly below (**D**,**E**). Graphs represent peptides which were only found in the absence or presence of gemcitabine (**C**–**E**). Statistical analysis was performed using the Mann–Whitney U test (GraphPad Prism, v.10.2.0). The asterisks indicate the following *p*-values: * *p* < 0.05, ** *p* < 0.01, *** *p* < 0.001, **** *p* < 0.0001. ^#^ Note that due to variable single, duplicate, or triplicate appearances of a peptide between replicates, the total number of shared peptides between No Treatment (757) and Gemcitabine (787) differed.

Based on our temperature-dependent assay findings and the knowledge that the quality (e.g., the affinity and immunogenicity) of presented peptide ligands is a critical determinant of HLA-I stability at the cell surface, we sought to determine what alterations were occurring at the peptide level. Following incubation in the presence or absence of gemcitabine, HLA-I-bound peptides were collected from S2-013 cells by mild acid elution (MAE), and eluted peptides were sequenced via mass spectrometry (Figure 7A). Differences between the immunopeptidomes of untreated and gemcitabine-exposed cells were summarized accordingly (Figure 7B–E).

Analysis of the mass spectrometry-identified sequences showed gemcitabine induced presentation of a population of unique peptides by HLA-I (Figure 7B). Allotype preferences and binding affinity for peptides, which were presented exclusively in the absence or presence of gemcitabine, were reported using NetMHCpan 4.1 (Figure 7C,D). The distribution of peptide binding among allotypes differed moderately following gemcitabine treatment (Figure 7C). However, peptide display for both treatment groups was dominated by HLA-A*02:06 and HLA-A*24:02, which accounted for presentation of nearly 90% of identified peptides (Figure 7C). Nonetheless, gemcitabine did not prevent the binding of peptides to any specific allotype expressed by this cell line, a critical observation since HLA haplotype expression among patients is diverse. Lists of the gemcitabine-exclusive peptide sequences and their matched allotype(s) are detailed (Tables S1 and S2). All putative binding peptides, regardless of treatment condition, were also reported (Table S3).

Although flexibility certainly exists, HLA-I molecules (and individual allotypes) maintain a proclivity for binding peptide ligands having certain lengths and amino acid residues [47]. We thus assessed differences in the length profile and carboxyl-terminal amino acid frequency for peptides which were unique to either the no treatment or gemcitabine-treatment groups. We observed a slight increase in the frequency of HLA-I-presented 9mers and a concomitant reduction in binding of 10mers and 12mers following treatment (Figure S1). These results suggest that gemcitabine improved the availability of peptide ligands that complemented the nonameric length preference of HLA-A2 and -A24 molecules [47]. Gemcitabine-exclusive peptides also demonstrated a shift in residue frequency at the C-terminus, a position which greatly influences binding affinity [48] (Figure S2). Decreased leucine and a slight upregulation of phenylalanine, isoleucine, and valine in the C-terminal position were noted, indicating gemcitabine’s favorable modulation of peptide binding (as these are the preferred C-terminal residues for HLA-A*02:06 and HLA-A*24:02) [49] (Figure S2).

In addition to our observations of general phenotypic properties, differences in peptide quality were also monitored. IC_50_ values reported with NetMHCpan 4.1 indicated that across all six allotypes, gemcitabine-exclusive peptides were stronger binders as evidenced by their reduced median IC_50_ [50] (Figure 7D). This improved binding affinity was also specific to peptides presented by HLA-A*02:06 molecules (Figure 7D). Although not statistically significant, HLA-A*24:02-displayed peptides followed a similar trend wherein gemcitabine treatment ameliorated predicted binding affinity (Figure 7D). A secondary measure of peptide quality is the ability to induce T cell recognition, or degree of immunogenicity. Immunogenicity scores for individual peptides were predicted via the IEDB immunogenicity modeling algorithm, which assigns a summation value to amino acids residing at specific positions within the peptide sequence [51]. The larger the immunogenicity score, the greater the expectation that a peptide can procure T cell reactivity [51]. Comparison of median immunogenicity scores between groups showed that gemcitabine-exclusive peptides were more immunogenic, regardless of their matched allotype (Figure 7E). In evaluating the dominant HLA-A allotypes, the median immunogenicity score of peptides presented by HLA-A*02:06 and HLA-A*24:02 after gemcitabine treatment also suggested heightened susceptibility for T cell recognition (Figure 7E). Consequently, it appears the gemcitabine exposure confers beneficial changes in the quality of peptides presented by HLA-I molecules.

### 2.4. Source Proteins which Generated HLA-I-Bound Peptides Differ after Gemcitabine Exposure in S2-013 Cells

**Figure 8:**
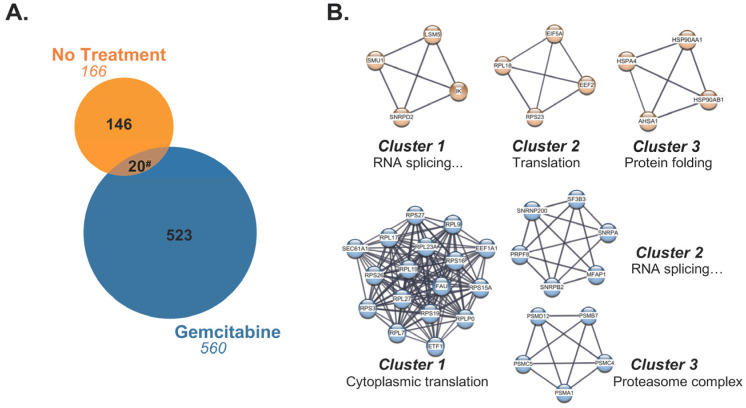
Gemcitabine-exclusive peptides are predominantly generated from distinctive source proteins. (**A**) Venn diagram displaying source proteins responsible for production of peptides exclusive to either the no treatment (orange) or gemcitabine-treated (blue) cohorts. Source proteins which gave rise to unique peptides but were shared between treatment groups (brown) are represented by the overlapping region. The numbers in italics indicate total number of source proteins. (**B**) The top three scoring clusters from the no treatment-exclusive (orange) and gemcitabine-exclusive (blue) source protein networks were identified with the MCODE plugin following network visualization in Cytoscape. Manually annotated gene ontology (GO) terms were assigned to each cluster using gProfiler’s enrichment analysis function. The GO term “RNA splicing via transesterification reactions with bulged adenosine as nucleophile” was abbreviated as “RNA splicing…” in the affected clusters. ^#^ Note that due to variable single, duplicate, or triplicate appearances of an exclusive peptide between replicates, the total number of shared source proteins between the no treatment-exclusive (20) and gemcitabine-exclusive (37) groups differed.

In line with the knowledge that gemcitabine facilitated HLA-I-presentation of a subset of unique peptides, we next evaluated the source proteins from which these exclusive peptides originated. Most of the gemcitabine-exclusive peptides were produced from distinctive source proteins which differed from those responsible for generation of no treatment-exclusive peptides (Figure 8A). To better understand the nature of these differences, we built a protein–protein interaction (PPI) network using the stringAPP in Cytoscape for source proteins of gemcitabine-exclusive peptides and compared it to the PPI network of source proteins that produced no treatment-exclusive peptides (Figure S3). The top three scoring clusters of highly interacting proteins were determined using the MCODE plugin in Cytoscape. Protein members of individual clusters were queried in gProfiler and assigned an ontology term that summarized their biological role (Figure 8B). Our results suggest that gemcitabine exposure perpetuates degradation of unique proteins with an increased prevalence of translation-related proteins, and that the peptide fragments of these proteins are suitable for HLA-I presentation (Figure 8B). Of note, there were approximately 20 source proteins shared by the treatment groups, indicating that gemcitabine either induced alternative cleavage patterns which resulted in unique peptide fragments or biased the loading of certain fragments over others (Figure 8A).

**Figure 9:**
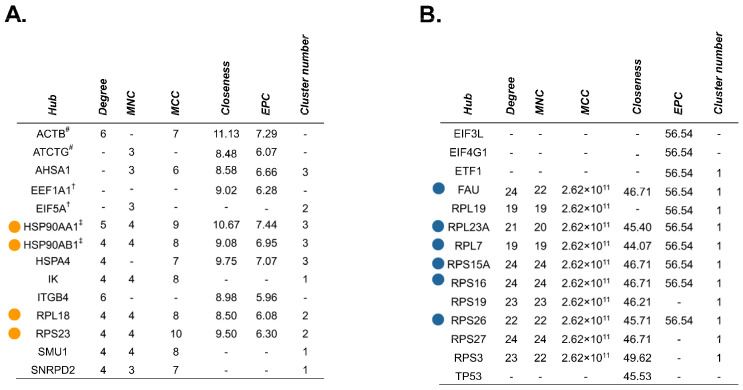
Unique hub proteins are identified in the source protein-protein interaction network following gemcitabine treatment. The cytoHubba plugin in Cytoscape was used to screen for hub proteins through five local- or global-based topological methods (Degree, MNC, MCC, Closeness, and EPC). Tables depict nodes classified as hubs by at least one of these methods in the no treatment-exclusive source protein network (**A**) and gemcitabine-exclusive source protein network (**B**). Colored dots represent candidate hub proteins reported by all five algorithms (**A**,**B**). Hubs matched to identical peptide sequences are denoted with symbols (^#^, ^†^, ^‡^) corresponding to their match (**A**).

The cytoHubba plugin identified critical nodes within the source protein networks using both local- (Degree, MNC, MCC) and global-based (Closeness, EPC) topological analysis methods [52]. Essential network proteins determined by at least one topological method were reported, and those which were mapped by all algorithms were classified as true hubs (Figure 9 and Figure S4). GO analysis designated two of the four identified hub proteins (HSP90AA1, HSP90AB1), as relating to RNA splicing for the no treatment-exclusive source protein–protein interaction network. The remaining two hub proteins (RPL18, RPS23), as well as all six hub proteins in the gemcitabine-exclusive source protein network (FAU, RPL23A, RPL7, RPS15A, RPS16, RPS26), appeared to be involved in translation and ribosome biogenesis. We also mapped the source protein networks via Ingenuity Pathway Analysis (IPA) and performed subsequent core pathway analyses (Figures S5 and S6). We identified eukaryotic initiation factor 2 (EIF2) pathway members as significant contributors to the immunopeptidome following gemcitabine treatment (Figure S6). The EIF2 signaling network plays an important role in regulating both global and specific mRNA translation [53]. Further enrichment analysis on the full source protein networks were also performed using gProfiler’s molecular function, biological process, and cellular component GO categories (Tables S4 and S5, Figures S7–S9). Our IPA results were corroborated by gene ontology analysis, with relevant GO terms implying overrepresentation of proteins involved in RNA (and DNA) binding after gemcitabine treatment (Table S5 and Figure S7). Of note, proteins based in the cytosol/cytoplasm, as well as cell motility and transport-related proteins, were common sources of peptides regardless of treatment status (Tables S4 and S5, Figures S8 and S9). These results reveal that while certain protein families regularly source peptides, gemcitabine treatment expands the repertoire of members which are degraded and ultimately presented by HLA-I molecules.

### 2.5. Gemcitabine Alters Immunoproteasome Catalytic Subunit Expression in Pancreatic Cancer Cells

**Figure 10:**
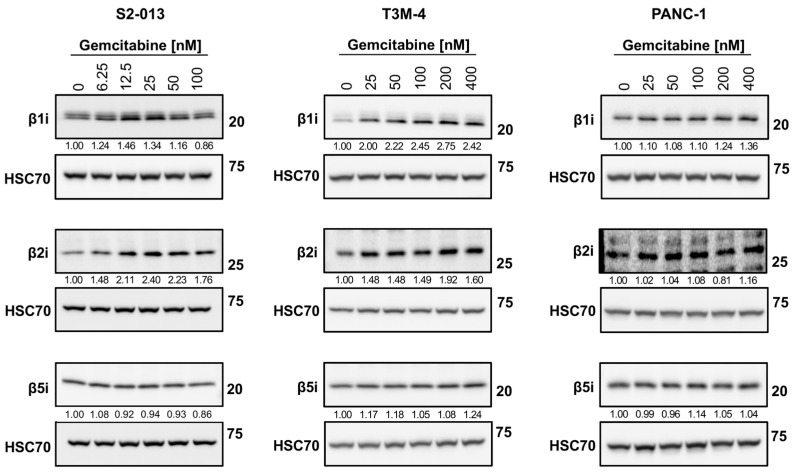
Gemcitabine increases expression of select immunoproteasome subunits in pancreatic cancer cells. S2-013, T3M-4, and PANC-1 cells were treated with various concentrations of gemcitabine for 24 h. Variations in total protein levels of the catalytic subunits of the immunoproteasome (β1i, β2i, and β5i) were assessed using western blots. HSC70 was used as a loading control. Blots are representative of at least three biological replicates. Numbers below bands indicate densitometric quantification performed with ImageLab (v. 6.1). Protein expression is relative first to the respective loading control and then normalized to the untreated sample.

Because the overall quality of the HLA-I-bound peptidome was enhanced by chemotherapy treatment, we speculated that gemcitabine favorably modulated peptide-generation mechanisms (in addition to altering the pool of source proteins). The composition of each peptide (e.g., affinity and immunogenicity) is initially influenced by its maker: the proteasome, a multi-subunit complex. In the presence of stimulating cytokines, the canonical proteasome’s catalytic subunits are outcompeted by the catalytic subunits of an inducible proteasome known as the immunoproteasome [21]. We monitored alterations in expression of the immunoproteasome catalytic subunits (β1i, β2i, and β5i) following gemcitabine exposure. In the S2-013 cells, certain concentrations of gemcitabine increased expression of the β1i catalytic subunit, and expression of the β2i immunosubunit was stimulated at all treatment concentrations (Figure 10). Increased protein expression of the β1i and β2i immunosubunits was observed at all gemcitabine concentrations in T3M-4 cells (Figure 10). Neither the β1i or β2i catalytic subunits were largely impacted by gemcitabine treatment in the PANC-1 cells, and gemcitabine did not modify protein expression of the third immunosubunit, β5i, in any of the tested cell lines (Figure 10). However, we note that β5i expression was largely maintained even in the presence of gemcitabine, preserving the potential to assemble fully formed immunoproteasomes. The immunoproteasome has been reported to generate more immunogenic peptides [23,24,54], and thus its induction may favorably modulate the HLA-I-bound peptidome.

## 3. Discussion

Pancreatic cancer is the third leading cause of cancer-related deaths in the United States [1], and the outlook for this disease remains poor. Although cytotoxic chemotherapy procures some success for patients, altered treatment strategies are desperately needed. A current investigational approach is identifying mechanisms which revitalize the anti-tumor immune response against pancreatic cancer. The success of immunotherapies (e.g., immune checkpoint inhibitors) in several types of cancer secures them as a viable option, but their current lack of clinical efficacy in the treatment of pancreatic cancer proposes the need for a suitable partner therapy [5,6,55]. Promising data have emerged from both animal studies and a preliminary clinical trial regarding the combination of gemcitabine with various types of immunotherapies [18,33,56]. In this study, we sought to uncover novel immunomodulatory activities of gemcitabine relating to its influence on tumor antigen recognition. We determined that gemcitabine treatment favorably altered regulation of HLA-I in pancreatic cancer, ultimately improving the likelihood for immune recognition.

Our results suggest that concentrations of gemcitabine which are cytostatic, but not overtly cytotoxic (Figure 1 and Figure 2), can modify tumor antigen presentation by pancreatic cancer cells. Antigens processed by one of several proteasomes are loaded onto HLA-I, and presentation of a peptide by an HLA-I molecule at the surface of the cell is used to facilitate cytotoxic T cell-mediated lysis. HLA-I downregulation is observed across many types of cancer, enabling tumor cell survival through mitigation of cytotoxic T cell responses [57]. In this study, we report that gemcitabine reinvigorated HLA-I expression in pancreatic cancer cells, and that its effects were observed at the mRNA, protein, and surface levels (Figure 2, Figure 3 and Figure 4). Contrary to previous results in lung, colon, and breast cancer cell lines [58], our data demonstrated gemcitabine upregulated transcripts of the light chain, (β_2_m), and heavy chains (HLA-A, HLA-B, and HLA-C), and that increases in both were also witnessed on the total protein level (more notably for HLA-B/C than HLA-A). Gemcitabine has previously been shown to enhance surface expression of HLA-I molecules in several cancer types including pancreatic [18,58,59], but our work provides insight on additional levels of regulation, as well as heavy chain and light chain-specific analyses that have not been addressed before.

It is likely that the alterations seen at the protein level result from transcriptional modifications, but the molecular mechanism by which gemcitabine increases mRNA transcripts of HLA-I-associated genes remains unexplored. A plausible explanation is that upon administration, gemcitabine instigates an anti-viral-like state in pancreatic cancer cells, perhaps through stimulation of inflammatory molecules and/or disruption of nucleotide pools. In this model, viral mimicry is induced by genotoxic insults, whereby aberrant DNA/RNA are detected via pattern recognition receptors (PRRs). Activation of PRRs enables production of proinflammatory cytokines, and through downstream signaling cascades, transcription of HLA-associated genes is augmented (e.g., through the binding of relevant interferon-sensitive response elements [ISRE] and nuclear factor kappa-light-chain-enhancer of activated B cells [NF-κB] sites located within these loci) [60,61]. In previous reports, gemcitabine induced micronuclei formation and secretion of inflammatory cytokines [18,62]. Thus, this could be a potential mechanistic route by which gemcitabine modulates HLA-I transcript and protein levels. Of note, a study in HeLa cells showed that gemcitabine’s activation of several interferon-stimulated genes was not reliant on interferon signaling and instead was mediated mostly by nucleotide availability [63]. Several other nucleotide-disrupting drugs have been shown to activate immune-associated genes, and there has been a growing body of evidence for chemotherapy-induced viral mimicry [64,65,66,67,68,69]. In the context of our research, it would be of further interest to assess whether increases in HLA-I-associated mRNA and protein expression are abrogated by inhibition of proinflammatory cytokine-mediated signaling or introduction of exogenous nucleotides.

Gemcitabine appeared to promote retainment of surface HLA-I molecules and modify the relative affinity of peptides available for binding (Figure 5 and Figure 6). Our hypothesis was confirmed by mass spectrometry sequencing of gemcitabine-treated S2-013 cells and downstream analysis on the immunopeptidome. Computational predictions indicated that gemcitabine-exclusive peptides were stronger binders and more immunogenic than no treatment-exclusive peptides (Figure 7D,E). Regardless of treatment status, we observed a bias towards isolation of HLA-A-binding peptides (Figure 7C). This finding was unexpected, and we reason it may be due to the HLA-I profile of the evaluated cell line. Surface HLA-A, -B, and -C molecules are not confined to 1:1:1 stoichiometry, and expression patterns can vary greatly among tissues and cell types [70,71,72,73,74]. It is plausible that the abundance of HLA-A2- and HLA-A24-binding peptides we reported was due to total surface outnumbering of these allotypes relative to their HLA-B/C counterparts. Though our mRNA and protein data did not overtly support such suspicions (Figure 2 and Figure 3), several post-translational mechanisms that impact the assembly of HLA-A, -B, and -C molecules can also contribute to differential surface expression [35,75,76,77]. For example, the structural motifs of HLA-A2 and HLA-A24 molecules render them susceptible to preferential “peptide editing” by a relevant chaperone protein over HLA-B/C allotypes [78], and this may explain their predominance in presentation of the isolated immunopeptidome in our experiments.

The diversity of HLA-I molecules is vast. Individual allotypes possess unique peptide-binding grooves [79], and these could vary in compatibility with the high quality antigens generated after gemcitabine treatment. However, our selection of the S2-013 cells for immunopeptidome analysis was intentional, as this cell line expresses common HLA-A molecules. HLA-A2 and HLA-A24 are highly prevalent allotypes possessed by ~30% and ~10% of the global population [34], respectively, and so we anticipate that our findings still retain important application for a large fraction of the population. In fact, the pancreatic cancer cell lines (S2-013, T3M-4, and PANC-1) used in this manuscript all express HLA-A2 (and two of the cell lines also share HLA-C12). However, the remainder of the HLA-I allotypes are unique among the three pancreatic cancer cell lines (Table S6), indicating that gemcitabine’s positive immunomodulatory effects are not entirely allotype-restricted and that they do apply to a range of HLA-I allotypes. Specifically, gemcitabine treatment increased mRNA levels, total protein expression, and the surface presence for diverse HLA-I allotype combinations (Figure 2, Figure 3 and Figure 4).

The presentation of a unique set of peptides by treated cells suggested gemcitabine procured changes in the canonical HLA-I antigen processing pathway. We investigated the impact of gemcitabine on immunoproteasome expression, a specialized proteasome induced by inflammatory cytokines (e.g., interferons). Important for viral clearance, the immunoproteasome characteristically generates higher quality peptides for HLA-I binding [21]. We found that certain concentrations of gemcitabine increased expression of the immunoproteasome catalytic subunits, albeit the magnitude of this increase varied between cell lines, and one of the immunosubunits, β5i, was not affected by treatment (Figure 10). This paralleled reported findings in several cancer cell lines after gemcitabine exposure [58], suggesting that while induction of the immunoproteasome may procure improvements in the presentation potential of HLA-I molecules (Figure 5 and Figure 7D,E), it is likely not the only mechanism responsible for gemcitabine’s modification of the immunopeptidome. Additionally, the assembly of hybrid proteasomes containing catalytic subunits from both the canonical proteasome and immunoproteasome have been described [80,81], and thus could generate the unique HLA-I-bound peptide population identified by mass spectrometry. Further studies should be performed to confirm if knockout of the immunosubunits, both individually and in tandem, negates gemcitabine-induced modulation of the HLA-I peptidome.

It is important to note that the proteasome, while ultimately responsible for peptide composition, is not the sole mediator of peptide selection. In conjunction with peptide processing by the immunoproteasome and/or hybrid proteasomes, it is possible that gemcitabine alters the proteome in pancreatic cancer cells and thus the proteins available for degradation. In this study, most gemcitabine-exclusive peptides originated from unique source proteins (Figure 8). Drug-induced variance in the protein landscape may explain that while source protein identity differed upon treatment status, conservation of protein families was retained. Abundant and high turnover proteins, such as ribosomal components, have been reported as common sources of peptides for HLA-I presentation [82], but we observed an apparent expansion of translation-associated source proteins following gemcitabine treatment (Table S5 and Figure S7). Notably, there were several ribosomal subunits within the gemcitabine-exclusive PPI network that were identified as essential hubs (Figure 9). It is plausible that the degradation of such translational machinery lies within gemcitabine’s pseudo-viral facade. Accordingly, activation of the translation-modifying integrated stress response (ISR) and prevention of protein synthesis machinery assembly are evident during anti-viral defense [83].

Our analyses were completed with pancreatic cancer cell lines rather than primary patient samples in order to observe transient gemcitabine-induced modifications in the immunopeptidome that are not generally present in patient autopsy samples (since most pancreatic cancer patients are not still receiving chemotherapy at the time of their deaths). An alternative approach would be to analyze tumor cells from gemcitabine-treated LSL-KrasG12D/+;LSL-Trp53R172H/+;Pdx-1-Cre (KPC) mice. However, a mouse model could not reflect the same diversity of HLA-I allotypes/immunopeptidomes expressed by humans, since mice express H-2 rather than HLA major histocompatibility complex molecules [84].

For our approach to immunopeptidome analysis, we selected mild acid elution (MAE) for isolation of HLA-I-bound peptides. MAE targets surface HLA-I-peptide complexes (most critical for T cell interaction), and it can also capture low affinity-binding peptides (in addition to higher affinity peptides) that are lost by other methods like immunoaffinity chromatography [85]. However, since MAE does not include HLA-I isolation as an interim step, there is a risk for concomitant collection of non-binding, contaminant peptides [85]. This risk was minimized using binding prediction models, although there is the caveat that the models would be influenced by the training data available [86].

Gemcitabine has previously been shown to increase pancreatic cancer cell migration, and expression of specific HLA-I allotypes were reported to differentially affect the motility of tumor cells [87,88,89,90]. Whether or not gemcitabine’s facilitation of migration is dependent on its stimulation of HLA-I was not explored in these publications nor our own. Gemcitabine has been approved as a monotherapy for pancreatic cancer since 1996 and used for several other types of cancer (as a monotherapy or in combination therapies) [91,92]. Moreover, use of gemcitabine in strategized combination therapies has demonstrated success in animal models [18,93]. These results suggest that even if the selected dosage of gemcitabine is associated with HLA-I-promoted cancer cell migration, the additional immuno-promotive events induced by gemcitabine still render the tumor susceptible to immune-mediated destruction.

We report an advantageous effect of gemcitabine exposure on pancreatic cancer cells: improvement in the quality of tumor peptide presentation. However, for this phenotype to perpetuate immune activity and hold biological significance, cytotoxic T cells must be present and active within the tumor microenvironment. Reports regarding gemcitabine’s influence on T cell infiltration vary. In a subcutaneous mouse model of pancreatic cancer, gemcitabine treatment inhibited infiltration of cytotoxic T cells [94], whereas in animal models of breast, lung, and (spontaneous) pancreatic cancer, tumor penetrance by T cells was enhanced by gemcitabine monotherapy or combination treatments containing gemcitabine [18,93,95]. Gemcitabine has been shown to reduce the presence of myeloid-derived suppressor cells (MDSCs) and regulatory T cells [14,96,97,98], and thus serves as a possible protective agent for effector T cell function. Accordingly, cytotoxic T cell proliferation rates have been reported to be maintained or enhanced following gemcitabine administration [14,98,99]. Of note, increased expression of programmed death-ligand 1 and 2 (PD-L1/PD-L2) and cytotoxic T lymphocyte-associated antigen 4 (CTLA-4) post-gemcitabine exposure have been observed [18,99,100,101]. Such immune checkpoints are under similar transcriptional regulation as HLA-I-associated genes [102], and so their expression is a likely byproduct of gemcitabine’s induction of proinflammatory cytokine secretion (e.g., IFNγ) [18,99].The dichotomy of gemcitabine’s immunomodulatory behaviors implies a need to mitigate immunosuppressive events while retaining desired promotive effects, likely possible through optimization of therapeutic partners [18,93].

Our collective observations implicate gemcitabine as a suitable agent for priming pancreatic cancer for immunotherapy-mediated destruction. Our results indicate that gemcitabine can improve the quality of tumor peptide presentation on pancreatic cancer cells, and of note, enhance the composition of the immunopeptidome. Though gemcitabine’s induction of immune checkpoints suggests the need for inclusion of relevant inhibitors [18,99,100,101], incorporation of anti-cancer vaccines remain another plausible partner therapy. The advancement of anti-cancer vaccines is a rapidly progressing field [103], and identification of novel epitopes induced by gemcitabine could be beneficial in the development of peptide-based vaccines for cancer treatment. A recent clinical trial combined chemotherapy, an immune checkpoint inhibitor (atezolizumab), and personalized vaccines for treatment of patients with resectable pancreatic cancer [104]. Although nearly half the patients demonstrated an immune response, vaccine targets were selected prior to chemotherapy administration [104]. However, our observed remodeling of the HLA-I peptidome by chemotherapy (i.e., gemcitabine) warrants the addition of drug-induced immunogenic epitopes during the vaccine development process. We reason the presence of chemotherapy-exclusive antigens likely will influence treatment scheduling regimens to ensure sufficient numbers of vaccine-specific T cells coincide with display of their peptide targets by HLA-I molecules.

## 4. Materials and Methods

### 4.1. Cell Lines and Culture Conditions

Human pancreatic cancer cell lines derived from both primary tumor (PANC-1) and metastases (T3M-4, S2-013) were used in this study. To specify, the T3M-4 cell line was obtained from a metastatic site in the lymph node, while the S2-013 line originated from a liver metastatic site. The S2-013 cell line is a sub-clone of the well-defined SUIT-2 cell line. The PANC-1 cell line was given by Dr. Michel Ouellette (University of Nebraska Medical Center, Omaha, NE, USA). The T3M-4 and S2-013 cell lines were donated by Dr. Angie Rizzino and Dr. Michael A. (Tony) Hollingsworth, respectively (University of Nebraska Medical Center, Omaha, NE, USA). Cell lines were confirmed to be mycoplasma-free using a PCR detection kit (Applied Biological Materials Inc., Richmond, BC, Canada).

The S2-013 cell line was cultured in supplemented Life Technologies RPMI 1640 (Thermo Fisher Scientific, Waltham, MA, USA), while the T3M-4 and PANC-1 cells were grown in supplemented Life Technologies DMEM (Thermo Fisher Scientific). Both forms of supplemented media contained identical additives: heat-inactivated (30 min, 56 °C) 10% fetal bovine serum, 10 mM HEPES, 2 mM L-glutamine, 1 mM sodium pyruvate, 1× non-essential amino acids, 100 units/mL penicillin, and 100 μg/mL streptomycin. All supplementation stocks were purchased from Thermo Fisher Scientific, apart from the fetal bovine serum, which was obtained from Atlantic Biologicals (Miami, FL, USA).

### 4.2. Antibodies and Drugs

For western blot analysis, individual components of the HLA-I complex were evaluated. Heavy chains were identified using an HLA-A heavy chain-specific antibody (#ab52922, Abcam, Cambridge, MA, USA) and the HC10 antibody which recognizes both HLA-B and HLA-C heavy chains. The light chain was detected through the use of an anti-β_2_m antibody (#ab75853, Abcam). For observation of immunoproteasome expression, antibodies against β1i (#14544-1-AP), β2i (#15976-1-AP), and β5i (#14859-1-AP) were used. All immunosubunit antibodies were purchased from Proteintech (Rosemont, IL, USA). Loading equality was verified through the housekeeping protein control antibody, HSC70 (#ADI-SPA-815-F, Enzo Life Sciences, Farmingdale, NY, USA) or GAPDH (2118S, Cell Signaling Technology, Danvers, MA, USA).

Individual components of the HLA-I complex were also identified by flow cytometry. BB7.2 was used to detect surface HLA-A2, one of the HLA-A heavy chains expressed by S2-013, T3M-4, and PANC-1 cells. An HLA-B/C antibody (B1.23.2, Thermo Fisher Scientific) was used for simultaneous, dual evaluation of all surface HLA-B and HLA-C heavy chains. For determination of conformationally specific and peptide-bound surface HLA-I, the W6/32 antibody was utilized. The HC10, BB7.2, and W6/32 antibodies were produced from hybridoma cell lines donated to us by Dr. Ted Hansen (Washington University, St. Louis, MO, USA).

Gemcitabine hydrochloride stock was purchased and diluted in water per experimentation requirements (#S1149, Selleckchem, Houston, TX, USA). Brefeldin A was obtained from Sigma-Aldrich (#B7651, St. Louis, MO, USA).

### 4.3. Proliferation and Viability Assays

#### 4.3.1. MTT

Cell proliferation was assessed using MTT experiments. Cell culture 96-well plates were seeded at pre-optimized densities (2500 cells/well for the S2-013 and T3M-4 cells, and 3000 cells/well for PANC-1), and cells were allowed to attach for 24 h (37 °C). Media containing various concentrations of gemcitabine were added to the wells. Following incubation in experimental media, MTT reagent (thiazolyl blue tetrazolium bromide, 98%; L11939, Alfa Aesar/Thermo Fisher Scientific) was dispensed into the wells for a final concentration of 0.5 mg/mL. The plates were incubated for an additional 3 h (37 °C), after which all liquid was removed. The remaining crystals were dissolved in isopropanol (300 μL), and plates were read at 570 nm with a SpectraMax M5e Microplate Reader (Molecular Devices, San Jose, CA, USA).

#### 4.3.2. Trypan Blue

Cell viability was evaluated using trypan blue staining. In brief, cells were seeded at a density of 500,000 cells/plate in 10 cm dishes. Cells were permitted to attach for 24 h, at which point they were administered gemcitabine or left untreated. Following incubation, aliquoted cells were mixed with 0.4% trypan blue stain (Invitrogen, Waltham, MA, USA) and counted on a hemocytometer. The percentage of viable cells was calculated as unstained cell number divided by total cell number × 100.

### 4.4. Flow Cytometry

TrypLE^TM^ Express Enzyme (Thermo Fisher) was used to remove plated cells. Following their dissociation, cells were resuspended in complete media and centrifuged (1500 rpm, 5 min, 4 °C) in an Eppendorf 5810R centrifuge (Eppendorf, Framingham, MA, USA). Upon pelleting, cells were diluted in FACS buffer (1× phosphate-buffered saline [PBS] containing 0.2% bovine serum albumin and 0.1% sodium azide) to a final concentration of 5 × 10^6^/mL and pipetted into a 96-well plate (100 μL/well). The plate was placed in an Eppendorf 5810R centrifuge and spun (1500 rpm, 5 min, 4 °C). Pelleted cells were resuspended in primary antibody and allowed to incubate (30 min, 4 °C). Following incubation, cells were centrifuged (1500 rpm, 5 min, 4 °C) and washed twice in 1× PBS. Fluorescently labeled secondary antibody was subsequently added and another incubation period ensued (30 min, 4 °C). The cells were then centrifuged (1500 rpm, 5 min, 4 °C), washed twice (1× PBS), and fixed in 1% paraformaldehyde. Analysis was performed on a BD LSR II Flow Cytometer (BD Biosciences, Franklin Lakes, NJ, USA) at the University of Nebraska Medical Center Flow Cytometry Research Facility. Data evaluation was conducted with FlowJo^TM^ v.10.9.

#### 4.4.1. Brefeldin A (BFA) Assay

For BFA flow cytometry experiments, cells were seeded in 10 cm dishes at 500,000 cells per dish and allowed 24 h to attach. Cells were either left untreated for control purposes or administered gemcitabine. Brefeldin A (2 mg/mL) was then added to the respective plates at pre-determined timepoints, and all cells were simultaneously harvested after conclusion of a 72-h treatment exposure. Cells were processed for flow cytometry as previously described.

#### 4.4.2. Temperature-Dependence Assay

For these flow cytometry assays, cells were seeded in 10 cm dishes at 500,000 cells per dish. Following a 24-h attachment period, experimental media containing the desired gemcitabine concentration were dispensed on the respective plates. Both untreated and treated plates were incubated at 37 °C. Designated low temperature plates were stored at 25 °C for at least 4 h prior to the conclusion of the incubation. All plates had a collective incubation period of 72 h. Cells were then harvested and prepared for flow cytometry as described in the protocol above.

### 4.5. Western Blotting

Plated cells were scraped and lysed using RIPA buffer (Thermo Fisher Scientific) supplemented with 2 mM DTT (Sigma), 1 mM Na_3_VO_4_ (Thermo Fisher Scientific), 0.1 mM phenylmethylsulfonyl fluoride (PMSF) (Sigma), and 1 μg/mL Halt Cocktail (Thermo Fisher Scientific). Lysates were stored at −80 °C. After thawing, lysates were centrifuged in an Eppendorf 5417R centrifuge (13,000 rpm, 30 min, 4 °C) and the supernatant was collected. The protein concentration for each sample was estimated via a bicinchoninic acid assay (BCA) kit (Thermo Fisher Scientific). Each lysate supernatant sample was combined with an appropriate volume of 5× sodium dodecyl sulfate loading dye, comprised of 10% *w*/*v* sodium dodecyl sulfate (Bio-Rad Laboratories, Hercules, CA, USA), 250 mM Tris-HCl pH 6.8, 30% *v*/*v* glycerol (Sigma), 0.2% *w*/*v* bromophenol blue (Sigma), and 5% *v*/*v* β-mercaptoethanol (Sigma). The samples were then boiled (5 min, 95 °C) and loaded into an Invitrogen Novex Tris-glycine polyacrylamide pre-cast gel (Thermo Fisher Scientific). The gel was electrophorized at 100 V for 2 h, after which the proteins were transferred onto a polyvinylidene difluoride Immobilon-P Millipore membrane for 1 h and 37 min at 30 V. The membrane was blocked to prevent non-specific binding in 5% *w*/*v* nonfat dry milk at room temperature, and primary antibodies were added. Following an overnight incubation at 4 °C, the membrane was washed at room temperature 3 times in 0.1% Tween-20 (Thermo Fisher Scientific) in Tris-buffered saline (TBS) pH 7.4, with washing periods of 15 min each. Appropriate secondary antibodies were placed on the membrane and incubation occurred at room temperature for a duration of 60 min. The membrane was then washed in 0.1% Tween-20 (Thermo Fisher Scientific) in Tris-buffered saline (TBS) pH 7.4 as previously described. Pierce ECL Western Blotting Substrate (Thermo Fisher Scientific) was dispensed on the membrane and incubation ensued (5 min, room temperature). The blot was analyzed using the Bio-Rad ChemiDoc Imaging System B and Image Lab software (v.6.1).

### 4.6. RNA Extraction and Reverse Transcription-Quantitative Polymerase Chain Reaction (RT-qPCR)

TRIzol^TM^ Reagent (Invitrogen, Thermo Fisher Scientific) was used to lyse plated cells. Scraping was performed and the cell/TRIzol^TM^ mixture was dispensed into 1.5-mL microcentrifuge tubes. In order to promote complete dissociation of nucleoprotein complexes, the homogenates were gently mixed and kept at room temperature for 5 min. Samples were then placed in the −80 °C freezer overnight. To extract the RNA, the homogenates were allowed to thaw on ice and then spun in an Eppendorf 5417R centrifuge (10,000 rpm, 10 min, 4 °C). Following centrifugation, the supernatants were collected and transferred into new microcentrifuge tubes and mixed with 200 µL chloroform. The samples were then shaken vigorously for 15 s and allowed to incubate at room temperature for 15 min to promote separation. The upper aqueous phase containing the desired RNA was transferred to a new tube and mixed with 250 µL of absolute ethanol (Decon Labs, King of Prussia, PA, USA). The remainder of the extraction procedure was conducted with the RNeasy Kit (Qiagen, Germantown, MD, USA) per manufacturer’s instructions. Resulting RNA concentration and purity was determined via a Nanodrop spectrophotometer (Thermo Fisher Scientific).

The collected RNA was then transformed into cDNA via the Affinity Script QPCR cDNA synthesis kit (Agilent Technologies, Santa Clara, CA, USA) in an MJ Research PTC-225 Peltier Thermal Cycler (MJ Research/Bio-Rad Laboratories, Hercules, CA, USA) and using the manufacturer’s suggested cycle settings (25 °C for 5 min, 42 °C for 15 min, and 95 °C for 5 min). The cDNA for each sample was added into its respective RT-qPCR reaction along with Applied Biosystems Power Up SYBR green master mix (Thermo Fisher Scientific) and 1 µM primer set, for a final volume of 25 µL. The utilized primers were specific for *HLA-A*, *HLA-B*, *HLA-C*, *B2M*, or the *GAPDH* control. The PrimerBank database (https://pga.mgh.harvard.edu/primerbank/, accessed on 17 July 2020) was used to generate primers, and their sequences are listed: *HLA-A* forward sequence 5′ ACCCTCGTCCTGCTACTCTC 3′ and reverse sequence 5′ CTGTCTCCTCGTCCCAATACT 3′; *HLA-B* forward sequence 5′ CAGTTCGTGAGGTTCGACAG 3′ and reverse sequence 5′ CAGCCGTACATGCTCTGGA 3′; *B2M* forward sequence 5′ GAGGCTATCCAGCGTACTCCA 3′ and reverse sequence 5′ CGGCAGGCATACTCATCTTTT 3′, and *GAPDH* forward sequence 5′ CTGGGCTACACTGAGCACC 3′ and reverse sequence 5′ AAGTGGTCGTTGAGGGCAATG 3′. Dr. Nicholas Mullen (University of Nebraska Medical Center, Omaha, NE, USA) kindly provided the *HLA-C* primers. *HLA-C* forward sequence 5’ GGACAAGAGCAGAGATACACG 3’ and reverse sequence 5’CAAGGACAGCTAGGACAACC 3’. The RT-qPCR was performed in the Applied Biosystems QuantStudio 3 Real-Time PCR System (Thermo Fisher Scientific) using the comparative Ct (ΔΔCt) method. Melting temperatures of the primers were used as a basis for determining thermal cycling conditions. Computation of the relative mRNA levels for each gene were calculated using the collected Ct values and the formula 2^−ΔCt^ for all primer sets.

### 4.7. HLA-I Peptide Elution, Mass Spectrometry, and Peptide Analysis

#### 4.7.1. Mild Acid Elution of HLA-Bound Peptides

Cells were seeded in 182-cm^2^ flasks (VWR International, Radnor, PA, USA) at a density of 3.125 × 10^6^ for a total collection value of 6.25 × 10^8^ cells per treatment group. Following a 72-h treatment in the presence or absence of gemcitabine, cells were collected using TrypLE^TM^ Express Enzyme (Thermo Fisher) and resuspended in complete media. Cells were kept on ice for the remainder of the protocol. Cell suspensions were then centrifuged in an Eppendorf 5810R centrifuge (211× *g*, 10 min, 4 °C) at which point the supernatant was removed and pelleted cells were resuspended in 1× PBS. This was repeated a second time. After removal of the supernatant, the cell pellet was gently mixed in 1 mL of mild acid elution (MAE) buffer for 1 min. The MAE buffer contained the following components: 131 mM citric acid (Sigma), 66 mM Na_2_HPO_4_ (Sigma), 150 mM NaCl (Sigma), 1 mM aprotinin (Thermo Fisher Scientific), and 25 mM iodoacetamide (Sigma). NaOH was used to adjust the MAE buffer to a pH of 3.3. Following resuspension in the buffer, the cell suspension was immediately centrifuged (285× *g*, 5 min, 4 °C), and the peptide-containing supernatant was collected. The peptide eluate was then centrifuged again (3375× *g*, 15 min, 4 °C) and further purified via ultracentrifugation (257,000× *g*, 60 min, 4 °C). Eluates were stored at −80 °C until further processing was required. This elution process was adapted from a previously published protocol [105].

#### 4.7.2. Mass Spectrometry Data Acquisition

The following procedure was conducted by MS Bioworks (Ann Arbor, MI, USA). Obtained peptide solutions were concentrated and desalted via solid-phase extraction (Waters µHLB C18 plate, Milford, MA, USA). Peptides were eluted with 80/20 acetonitrile/water (0.1% TFA), lyophilized, and then reconstituted in 0.1% TFA. Following purification, peptides (50% per sample) were evaluated with a nano LC/MS/MS on a Waters NanoAcquity system coupled to a Thermo Fisher Fusion Lumos mass spectrometer. Samples were loaded onto a trapping column and separated on an analytical column (75 µm, 350 nL/min), both of which were packed with XSelect C18 (Waters). A 2 h gradient was used. Peptide sequencing was performed using a custom data-dependent method. Intact peptides were detected in the Orbitrap at a resolution of 60,000 FWHM. Sequential MS/MS was performed in the Orbitrap at 15,000 FWHM using high resolution collision-induced dissociation and electron transfer/higher energy collisional dissociation. All MS steps employed cycle times of 3 s and a scan range of *m*/*z* 300–800. The resulting data were processed with a local copy of PEAKS Studio (v.10.6).

Peptide elution and MS analysis was completed on paired no treatment and gemcitabine-treated samples from three independent experiments. The data presented in this manuscript represent the pooled results of these experiments. Only peptides which retained true exclusivity status among the replicates were categorized as such and included in subsequent analyses.

#### 4.7.3. Identification of Putative Binders and Assessment of Peptides’ Features

Sequenced peptides were further siphoned based on length (8–12 amino acids) to ensure the likelihood of HLA-I binding, and were classified into two groups: peptides identified only from the control cells (no treatment-exclusive) and peptides unique to gemcitabine-treated cells (gemcitabine-exclusive). The resulting peptide list was input into the NetMHCpan 4.1 database (https://services.healthtech.dtu.dk/services/NetMHCpan-4.1/, accessed on 10 June 2023) to predict binding compatibility with the HLA-I allotypes expressed by the analyzed cell line (Table S6). Binding affinity (IC_50_, nM) was reported by NetMHCpan 4.1. Downstream peptide analysis was only performed on peptides which were modeled to bind with a NetMHC IC_50_ of ≤500 nM and thus expected putative binders of HLA-I molecules. An inverse relationship exists between the IC_50_ value and strength of peptide/HLA-I association, wherein lower IC_50_ values indicate higher affinity [50,106]. Of note, the existence of similar binding preferences between allotypes permitted some peptides to be matched to multiple HLA-I molecules. In these circumstances, the allotype/peptide pairing which exhibited the strongest affinity was selected for inclusion in all summary data.

Immunogenicity scores for individual peptides were estimated via the Immune Epitope Database (IEDB) Next Generation Tools-Class I pMHC Immunogenicity prediction model (https://nextgen-tools.iedb.org/pipeline, accessed on 23 June 2023). The anchor masking feature of this tool improves the validity of the calculated immunogenicity score by excluding residues that primarily mediate binding to the HLA-I molecule and not direct T cell interaction [51]. Masked residues included the 1st, 2nd, and C-terminal positions for the HLA-A*02:06, -A*24:02, -B*07:02, -B*59:01, and -C*07:02 allotypes [107]. For peptides which bound HLA-C*01:02, anchor residues at the 2nd, 3rd, and C-terminal positions were excluded [108]. A correlated relationship exists between the calculated score and the potential for T cell recognition, wherein the more positive the immunogenicity score, the more likely T cell reactivity will occur [51].

#### 4.7.4. Evaluation of Source Proteins

Mass spectrometry-sequenced peptides were matched to their respective protein of origin, herein referred to as source proteins, via the PEAKS Studio software (v.10.6) described above. Some peptides were mapped to multiple source proteins, most often a result of conserved amino acid sequences within protein families. Because it is plausible that the degradation of several proteins produced identical peptide ligands, we included all mapped source proteins in relevant analyses. Full length protein names were retrieved by inputting the PEAKS-assigned IDs into the UniProt Retrieve/ID mapping tool (https://www.uniprot.org/id-mapping, accessed on 17 September 2023).

Source proteins which generated either no treatment-exclusive peptides or gemcitabine-exclusive peptides were queried with the stringAPP (v. 2.0.1) and displayed in Cytoscape (v. 3.10.1) to build a protein–protein interaction (PPI) network. The physical subnetwork was retrieved with high confidence (score > 0.7). Prominent protein clusters were identified via MCODE (default settings, v. 2.0.3) and visualized on the full network with AutoAnnotate (v. 1.4.1). An ontology term (biological process) was manually assigned to each cluster following gProfiler (v. e109_eg56_p17_1d3191d) analysis (see below for inclusion criteria).

Network-wide enrichment evaluation was also conducted with gProfiler. Source proteins which generated no treatment-exclusive or gemcitabine-exclusive peptides were queried against the “GO-molecular function”, “GO-biological process”, and “GO-cellular component” databases using the default background list. Overrepresented driver terms were reported (Tables S4 and S5, Figures S7–S9), and the *p*-value assigned to each term indicates its corrected significance (g:SCS method). Network-wide mapping was also performed with Ingenuity Pathway Analysis (IPA, v.23.0). Core analyses were conducted using the following parameters: high confidence and experimentally observed associations, direct interactions, human species (Figures S5 and S6).

Nodes within the PPI network were ranked via the cytoHubba plugin (v.0.1). Five local-(Degree, Maximum Neighborhood Component [MNC], Maximal Clique Centrality [MCC]) and global-based (Closeness, Edge Percolated Component [EPC]) topological methods were employed to define the top 10 essential proteins within the network [52]. Identified hub proteins were input into a Venn diagram generator (https://bioinformatics.psb.ugent.be/webtools/Venn/, accessed 7 November 2023) to evaluate common hubs reported by all five algorithms. Inkscape (v.1.2) was used for further figure customization (Figure S4).

Venn diagrams used to display no treatment-exclusive and gemcitabine-exclusive source proteins/peptides were created with BioVenn (https://www.biovenn.nl/, accessed 16 October 2023) and recolored in PowerPoint (v.16.82). Of additional note, there were certain source proteins/peptides which were identified multiple times. Because their frequency may have differed (e.g., found in 2/3 control samples, but 3/3 gemcitabine samples), the total source protein/peptide values differed between treatment groups. This has been indicated by a “#” in the affected Venn diagrams.

## 5. Conclusions

In conclusion, our studies highlight the immunomodulatory capabilities of the chemotherapy drug gemcitabine, relating to its favorable regulation of tumor antigen presentation. Specifically, we provide novel evidence that gemcitabine augments expression of the immunostimulatory complex human leukocyte antigen class I (HLA-I), and it also improves the quality of peptide ligands presented by these molecules by pancreatic cancer cells. The display of peptide fragments from abnormal proteins and activation of T cell-mediated immunity is a critical component of the adaptive immune response. Our work thus has applications beyond cancer, and could be important in improving patient outcomes for other relevant medical conditions in which the modulation of HLA-I regulation using standard care treatments remains largely unexplored.

## Data Availability

The original contributions presented in the study are included in the article/Supplementary Materials; further inquiries can be directed to the corresponding author/s.

## Supplementary Materials

The following supporting information can be downloaded at: https://www.mdpi.com/article/10.3390/ijms25063211/s1.
