# Supplementary material for: Gemcitabine Modulates HLA-I Regulation to Improve Tumor Antigen Presentation by Pancreatic Cancer Cells
Source: Int J Mol Sci. 2024 Mar 11;25(6):3211. doi: 10.3390/ijms25063211 (PMC10970070; doi:10.3390/ijms25063211)
Supplement: Supplementary file 1 [file ijms-25-03211-s001.zip › Supplementary Figures S1-S9.pptx]

## Slide 1
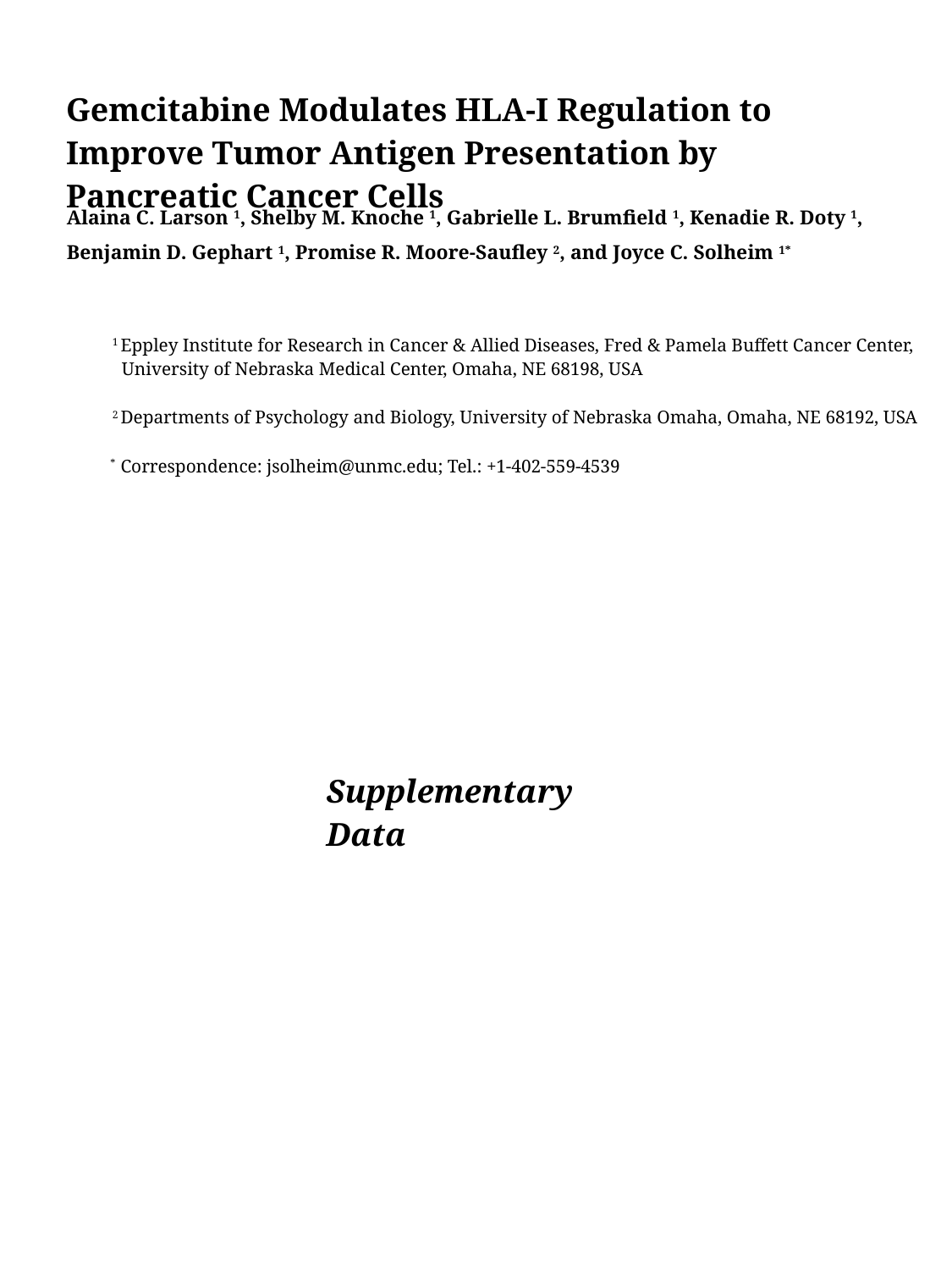

| Gemcitabine Modulates HLA-I Regulation to Improve Tumor Antigen Presentation by Pancreatic Cancer Cells |
| --- |
Alaina C. Larson 1, Shelby M. Knoche 1, Gabrielle L. Brumfield 1, Kenadie R. Doty 1,
Benjamin D. Gephart 1, Promise R. Moore-Saufley 2, and Joyce C. Solheim 1*
| |
| --- |
| |
| 1 Eppley Institute for Research in Cancer & Allied Diseases, Fred & Pamela Buffett Cancer Center, University of Nebraska Medical Center, Omaha, NE 68198, USA |
| 2 Departments of Psychology and Biology, University of Nebraska Omaha, Omaha, NE 68192, USA |
| \*  Correspondence: jsolheim@unmc.edu; Tel.: +1-402-559-4539 |
| Supplementary Data |
| --- |

## Slide 2
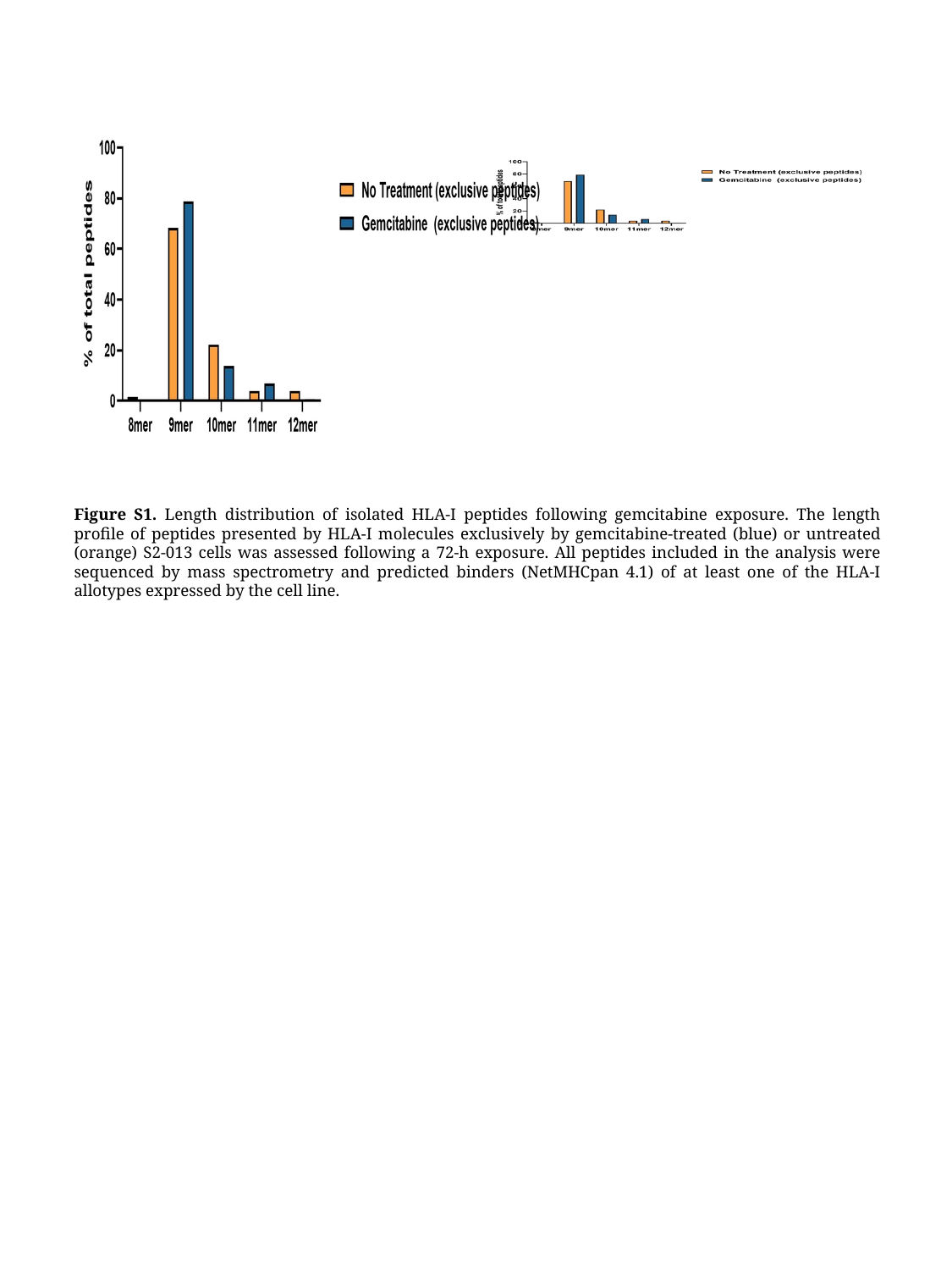

Figure S1. Length distribution of isolated HLA-I peptides following gemcitabine exposure. The length profile of peptides presented by HLA-I molecules exclusively by gemcitabine-treated (blue) or untreated (orange) S2-013 cells was assessed following a 72-h exposure. All peptides included in the analysis were sequenced by mass spectrometry and predicted binders (NetMHCpan 4.1) of at least one of the HLA-I allotypes expressed by the cell line.

## Slide 3
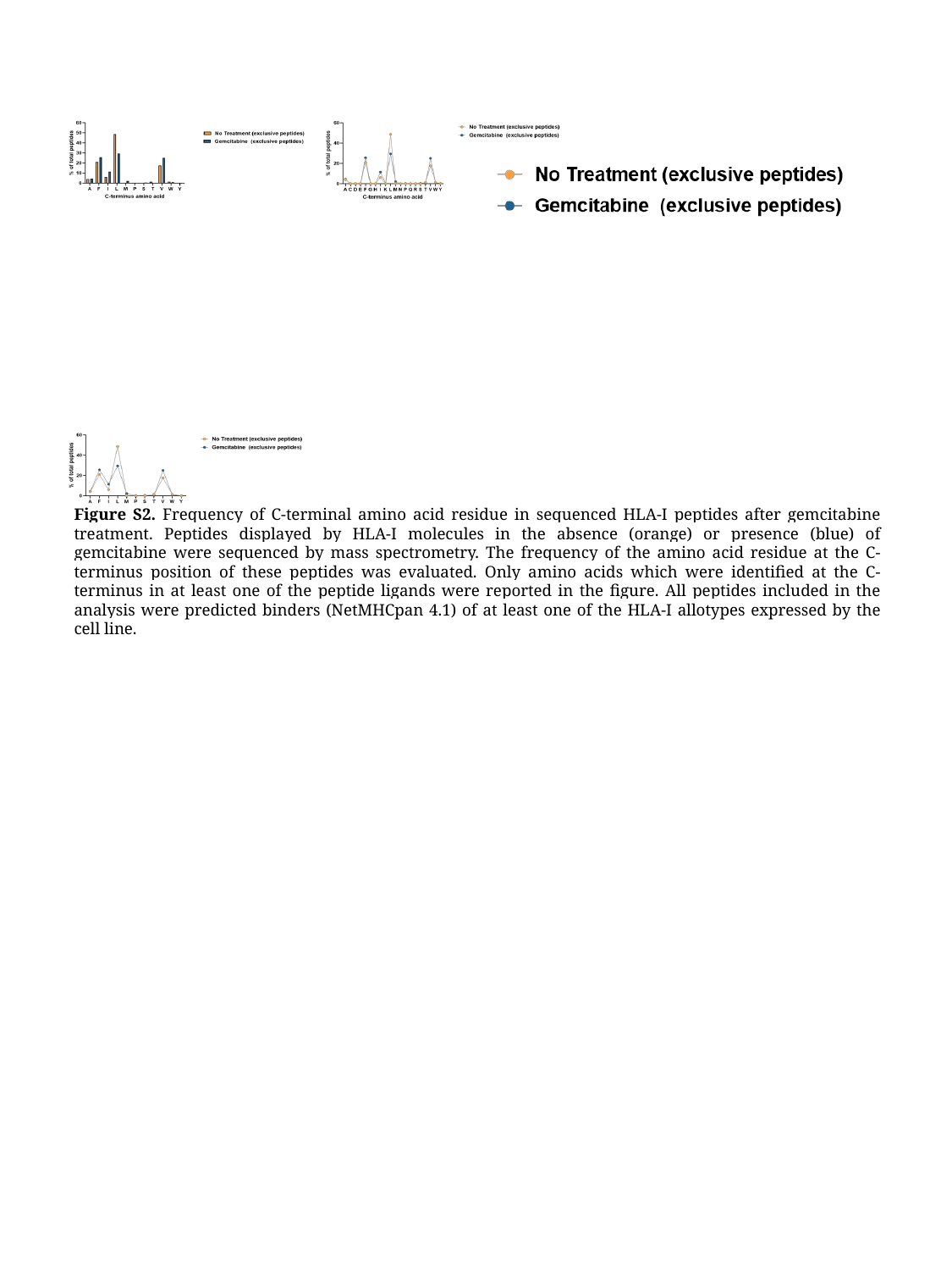

Figure S2. Frequency of C-terminal amino acid residue in sequenced HLA-I peptides after gemcitabine treatment. Peptides displayed by HLA-I molecules in the absence (orange) or presence (blue) of gemcitabine were sequenced by mass spectrometry. The frequency of the amino acid residue at the C-terminus position of these peptides was evaluated. Only amino acids which were identified at the C-terminus in at least one of the peptide ligands were reported in the figure. All peptides included in the analysis were predicted binders (NetMHCpan 4.1) of at least one of the HLA-I allotypes expressed by the cell line.

## Slide 4
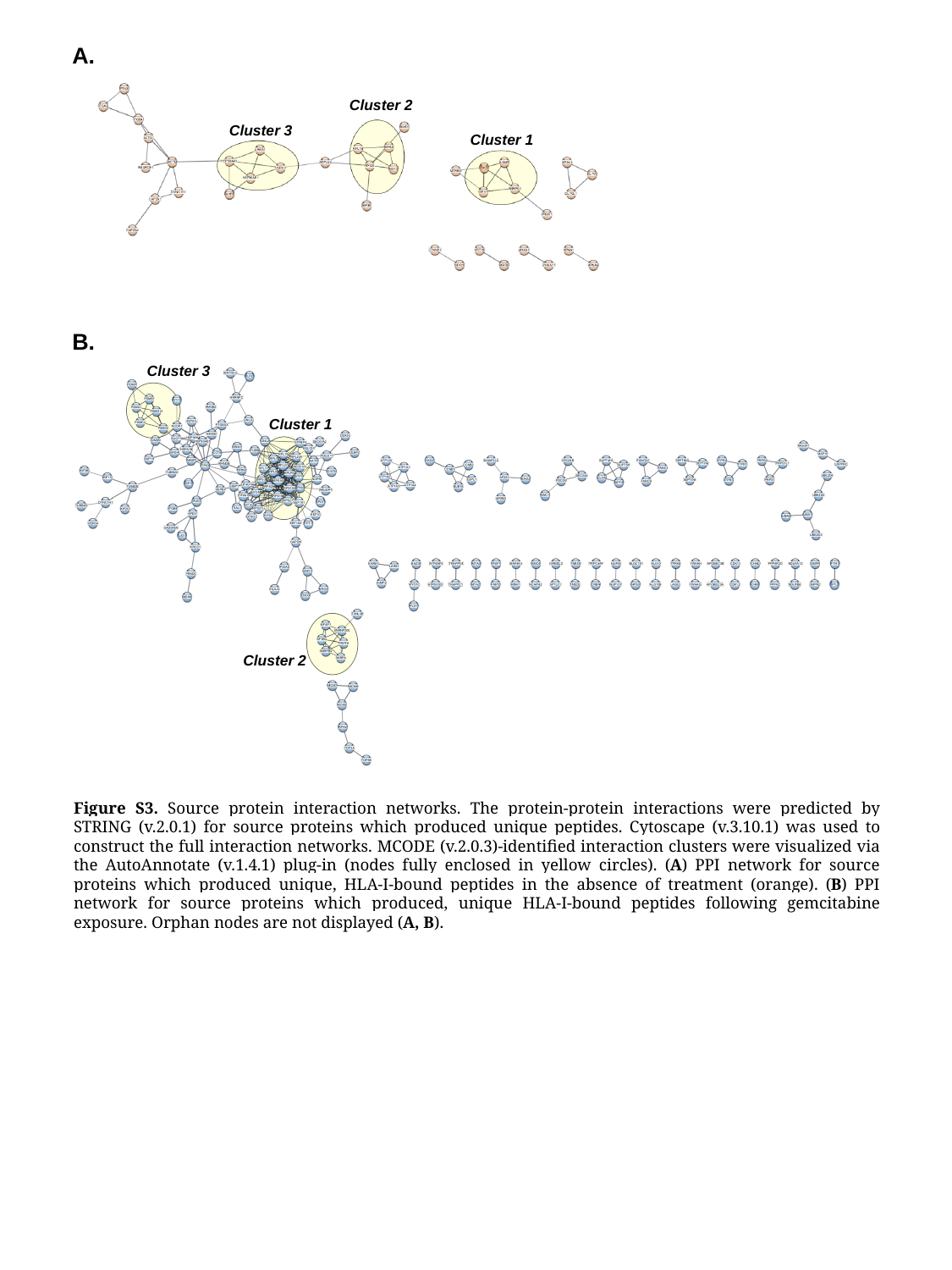

A.
Cluster 2
Cluster 3
Cluster 1
B.
Cluster 3
Cluster 1
Cluster 2
Figure S3. Source protein interaction networks. The protein-protein interactions were predicted by STRING (v.2.0.1) for source proteins which produced unique peptides. Cytoscape (v.3.10.1) was used to construct the full interaction networks. MCODE (v.2.0.3)-identified interaction clusters were visualized via the AutoAnnotate (v.1.4.1) plug-in (nodes fully enclosed in yellow circles). (A) PPI network for source proteins which produced unique, HLA-I-bound peptides in the absence of treatment (orange). (B) PPI network for source proteins which produced, unique HLA-I-bound peptides following gemcitabine exposure. Orphan nodes are not displayed (A, B).

## Slide 5
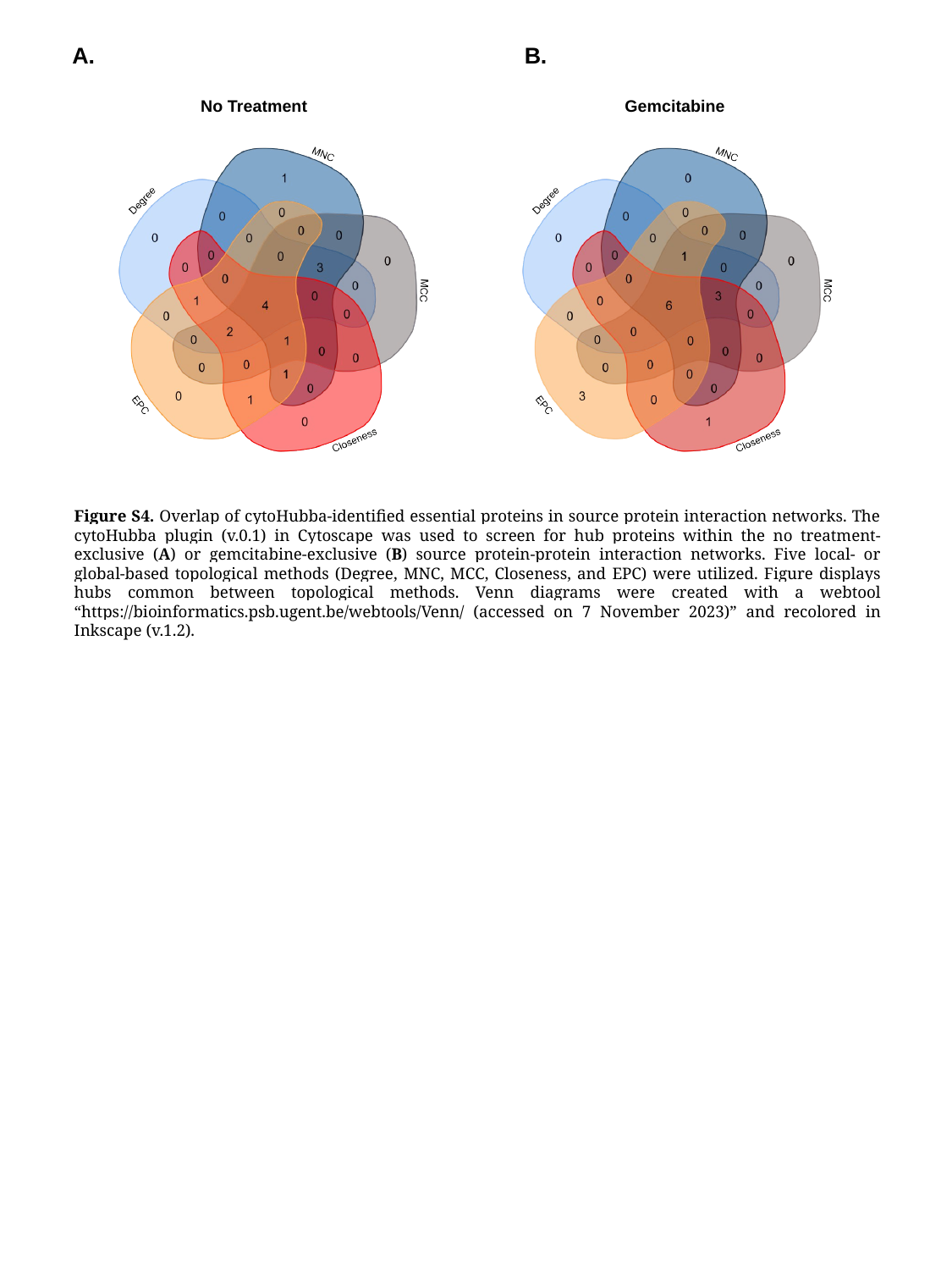

A.
B.
No Treatment
Gemcitabine
Figure S4. Overlap of cytoHubba-identified essential proteins in source protein interaction networks. The cytoHubba plugin (v.0.1) in Cytoscape was used to screen for hub proteins within the no treatment-exclusive (A) or gemcitabine-exclusive (B) source protein-protein interaction networks. Five local- or global-based topological methods (Degree, MNC, MCC, Closeness, and EPC) were utilized. Figure displays hubs common between topological methods. Venn diagrams were created with a webtool “https://bioinformatics.psb.ugent.be/webtools/Venn/ (accessed on 7 November 2023)” and recolored in Inkscape (v.1.2).

## Slide 6
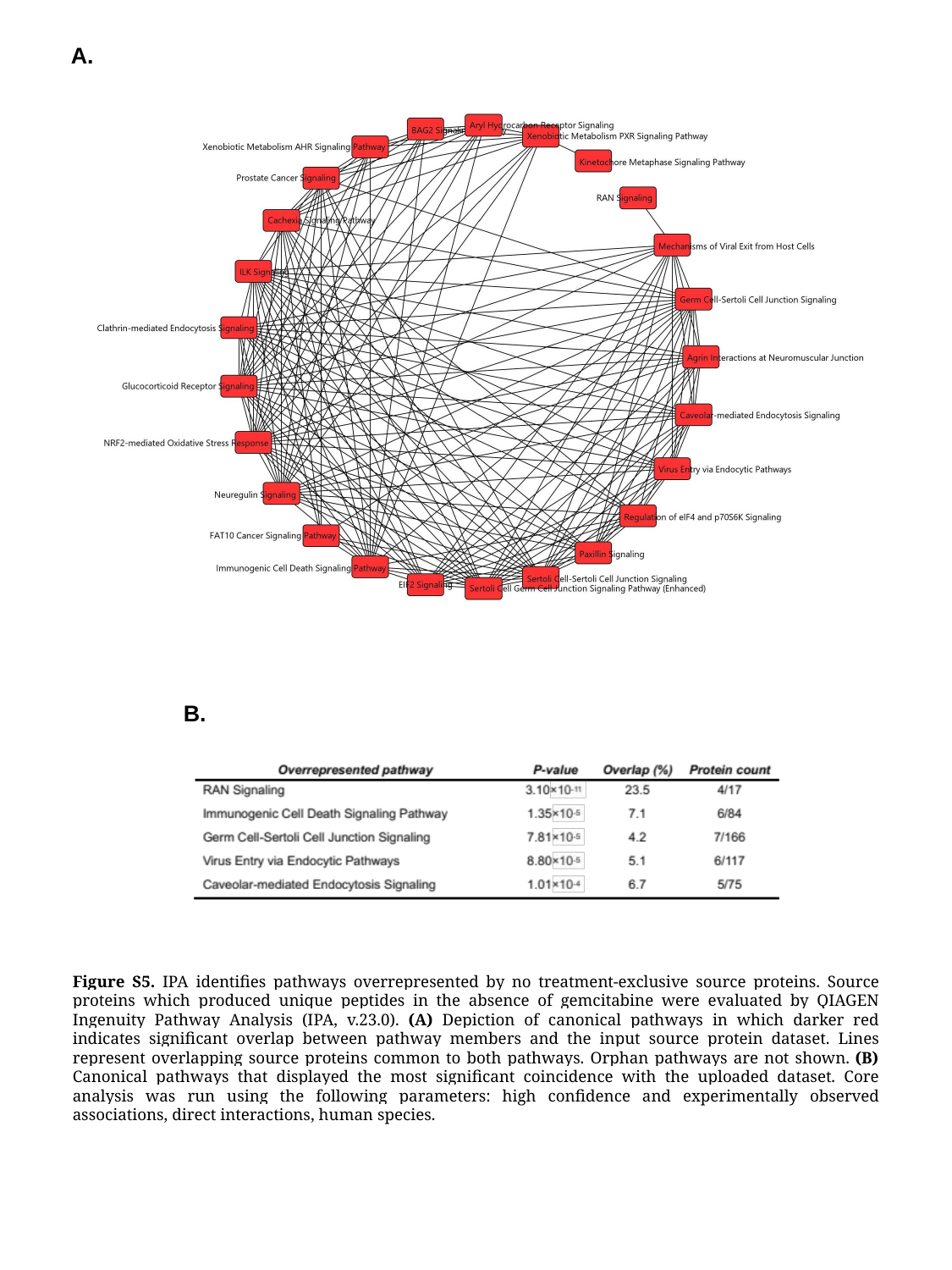

A.
B.
Figure S5. IPA identifies pathways overrepresented by no treatment-exclusive source proteins. Source proteins which produced unique peptides in the absence of gemcitabine were evaluated by QIAGEN Ingenuity Pathway Analysis (IPA, v.23.0). (A) Depiction of canonical pathways in which darker red indicates significant overlap between pathway members and the input source protein dataset. Lines represent overlapping source proteins common to both pathways. Orphan pathways are not shown. (B) Canonical pathways that displayed the most significant coincidence with the uploaded dataset. Core analysis was run using the following parameters: high confidence and experimentally observed associations, direct interactions, human species.

## Slide 7
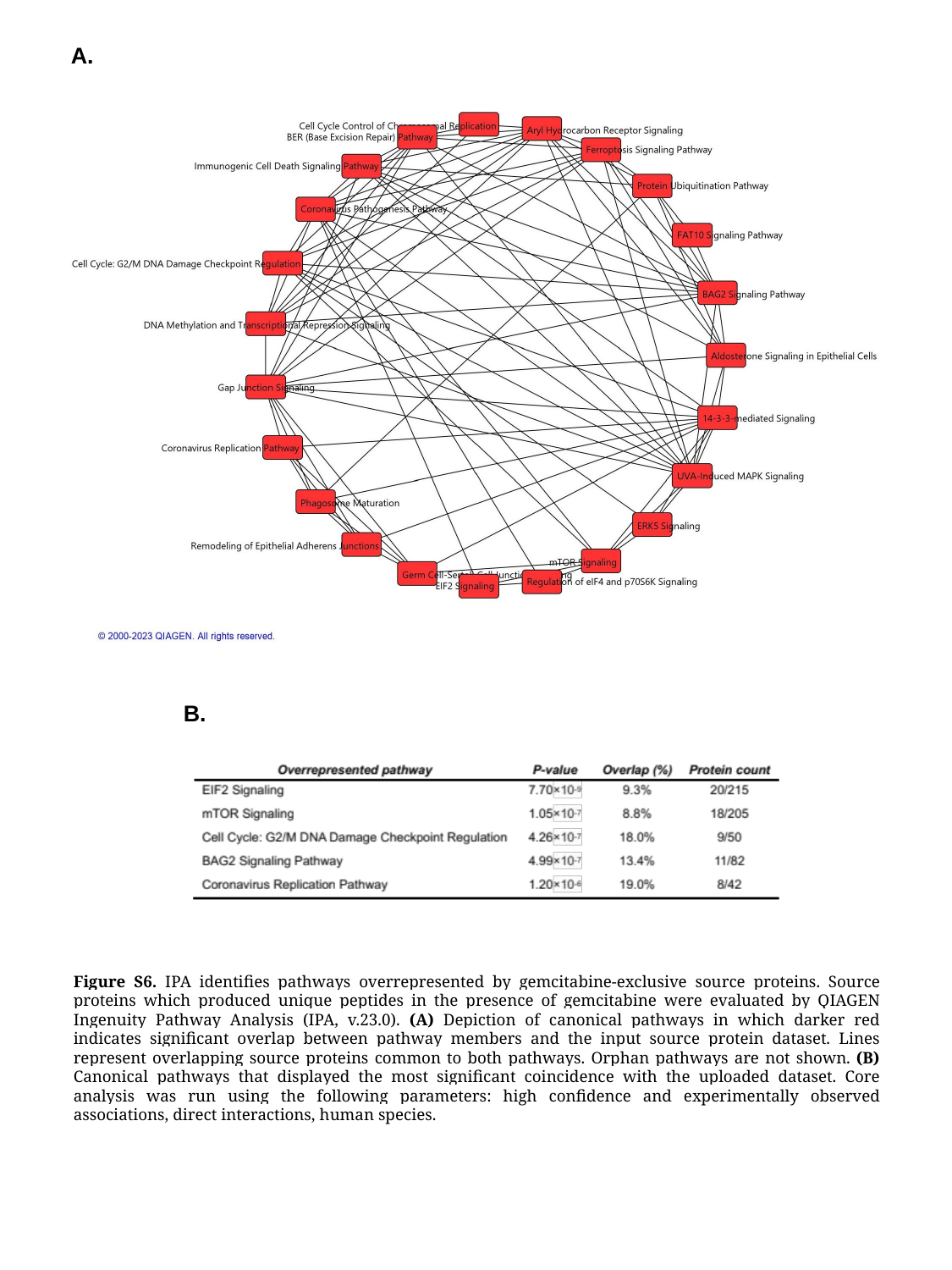

A.
B.
Figure S6. IPA identifies pathways overrepresented by gemcitabine-exclusive source proteins. Source proteins which produced unique peptides in the presence of gemcitabine were evaluated by QIAGEN Ingenuity Pathway Analysis (IPA, v.23.0). (A) Depiction of canonical pathways in which darker red indicates significant overlap between pathway members and the input source protein dataset. Lines represent overlapping source proteins common to both pathways. Orphan pathways are not shown. (B) Canonical pathways that displayed the most significant coincidence with the uploaded dataset. Core analysis was run using the following parameters: high confidence and experimentally observed associations, direct interactions, human species.

## Slide 8
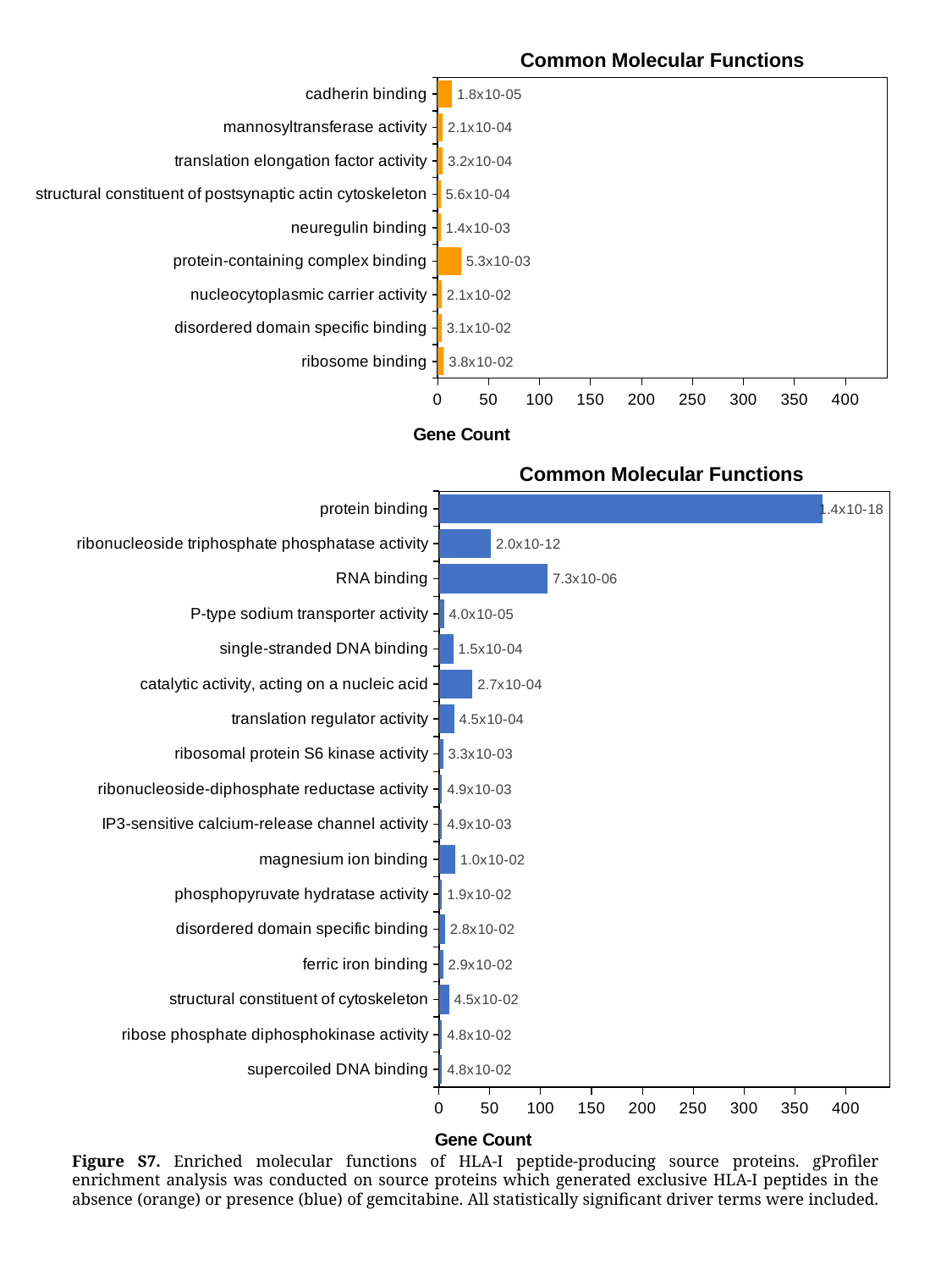

### Chart: Common Molecular Functions
| Category | |
|---|---|
| cadherin binding | 14.0 |
| mannosyltransferase activity | 5.0 |
| translation elongation factor activity | 5.0 |
| structural constituent of postsynaptic actin cytoskeleton | 3.0 |
| neuregulin binding | 3.0 |
| protein-containing complex binding | 23.0 |
| nucleocytoplasmic carrier activity | 4.0 |
| disordered domain specific binding | 4.0 |
| ribosome binding | 6.0 |Common Molecular Functions
### Chart: Common Molecular Functions
| Category | |
|---|---|
| protein binding | 377.0 |
| ribonucleoside triphosphate phosphatase activity | 51.0 |
| RNA binding | 107.0 |
| P-type sodium transporter activity | 5.0 |
| single-stranded DNA binding | 14.0 |
| catalytic activity, acting on a nucleic acid | 33.0 |
| translation regulator activity | 15.0 |
| ribosomal protein S6 kinase activity | 4.0 |
| ribonucleoside-diphosphate reductase activity | 3.0 |
| IP3-sensitive calcium-release channel activity | 3.0 |
| magnesium ion binding | 16.0 |
| phosphopyruvate hydratase activity | 3.0 |
| disordered domain specific binding | 6.0 |
| ferric iron binding | 4.0 |
| structural constituent of cytoskeleton | 10.0 |
| ribose phosphate diphosphokinase activity | 3.0 |
| supercoiled DNA binding | 3.0 |Common Molecular Functions
Figure S7. Enriched molecular functions of HLA-I peptide-producing source proteins. gProfiler enrichment analysis was conducted on source proteins which generated exclusive HLA-I peptides in the absence (orange) or presence (blue) of gemcitabine. All statistically significant driver terms were included.

## Slide 9
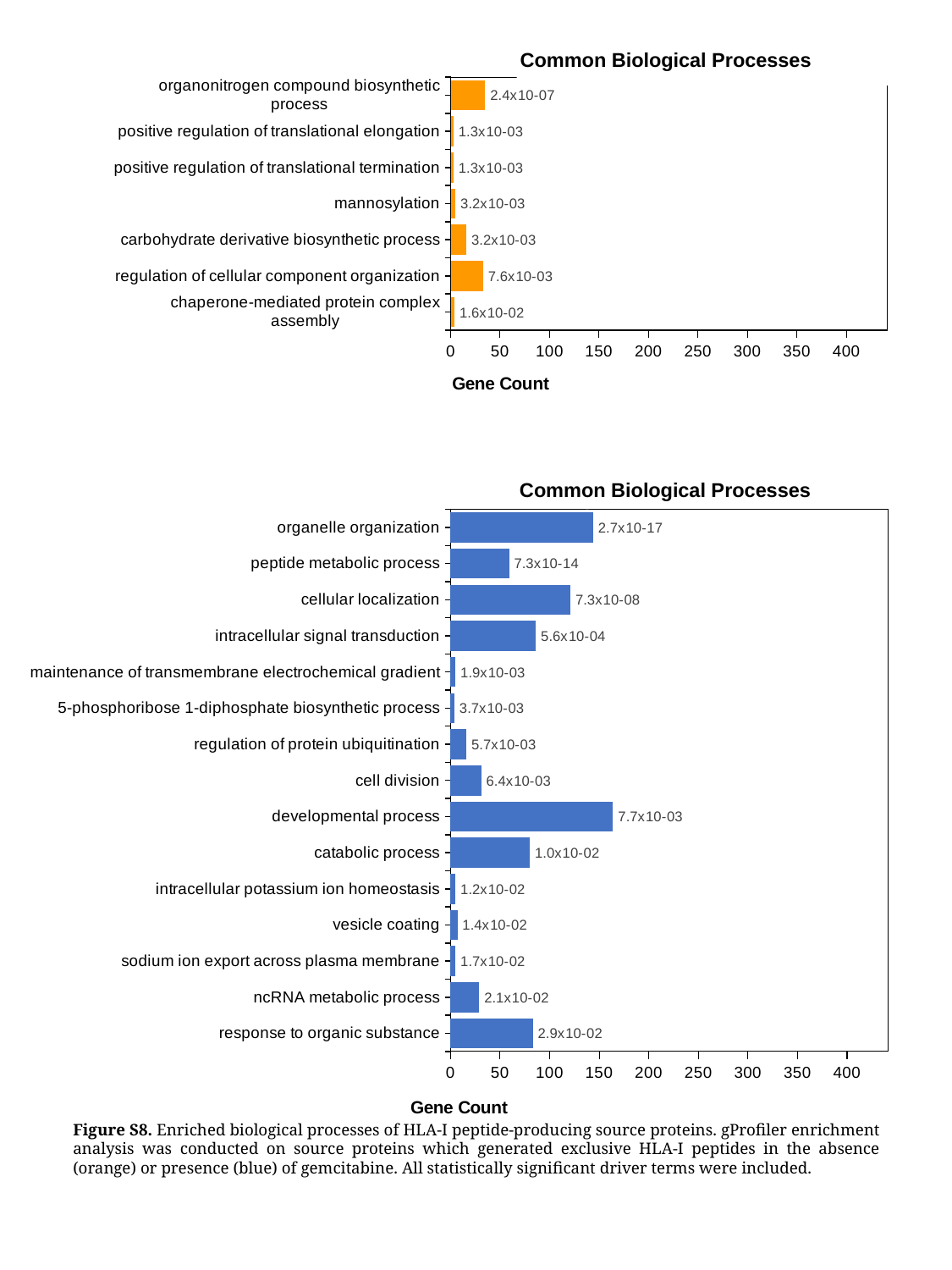

### Chart: Common Biological Processes
| Category | |
|---|---|
| organonitrogen compound biosynthetic process | 35.0 |
| positive regulation of translational elongation | 3.0 |
| positive regulation of translational termination | 3.0 |
| mannosylation | 5.0 |
| carbohydrate derivative biosynthetic process | 16.0 |
| regulation of cellular component organization | 33.0 |
| chaperone-mediated protein complex assembly | 4.0 |Common Biological Processes
### Chart: Biological Processes
| Category | |
|---|---|
| organelle organization | 144.0 |
| peptide metabolic process | 59.0 |
| cellular localization | 121.0 |
| intracellular signal transduction | 86.0 |
| maintenance of transmembrane electrochemical gradient | 5.0 |
| 5-phosphoribose 1-diphosphate biosynthetic process | 4.0 |
| regulation of protein ubiquitination | 16.0 |
| cell division | 31.0 |
| developmental process | 164.0 |
| catabolic process | 80.0 |
| intracellular potassium ion homeostasis | 5.0 |
| vesicle coating | 7.0 |
| sodium ion export across plasma membrane | 5.0 |
| ncRNA metabolic process | 29.0 |
| response to organic substance | 83.0 |Common Biological Processes
Figure S8. Enriched biological processes of HLA-I peptide-producing source proteins. gProfiler enrichment analysis was conducted on source proteins which generated exclusive HLA-I peptides in the absence (orange) or presence (blue) of gemcitabine. All statistically significant driver terms were included.

## Slide 10
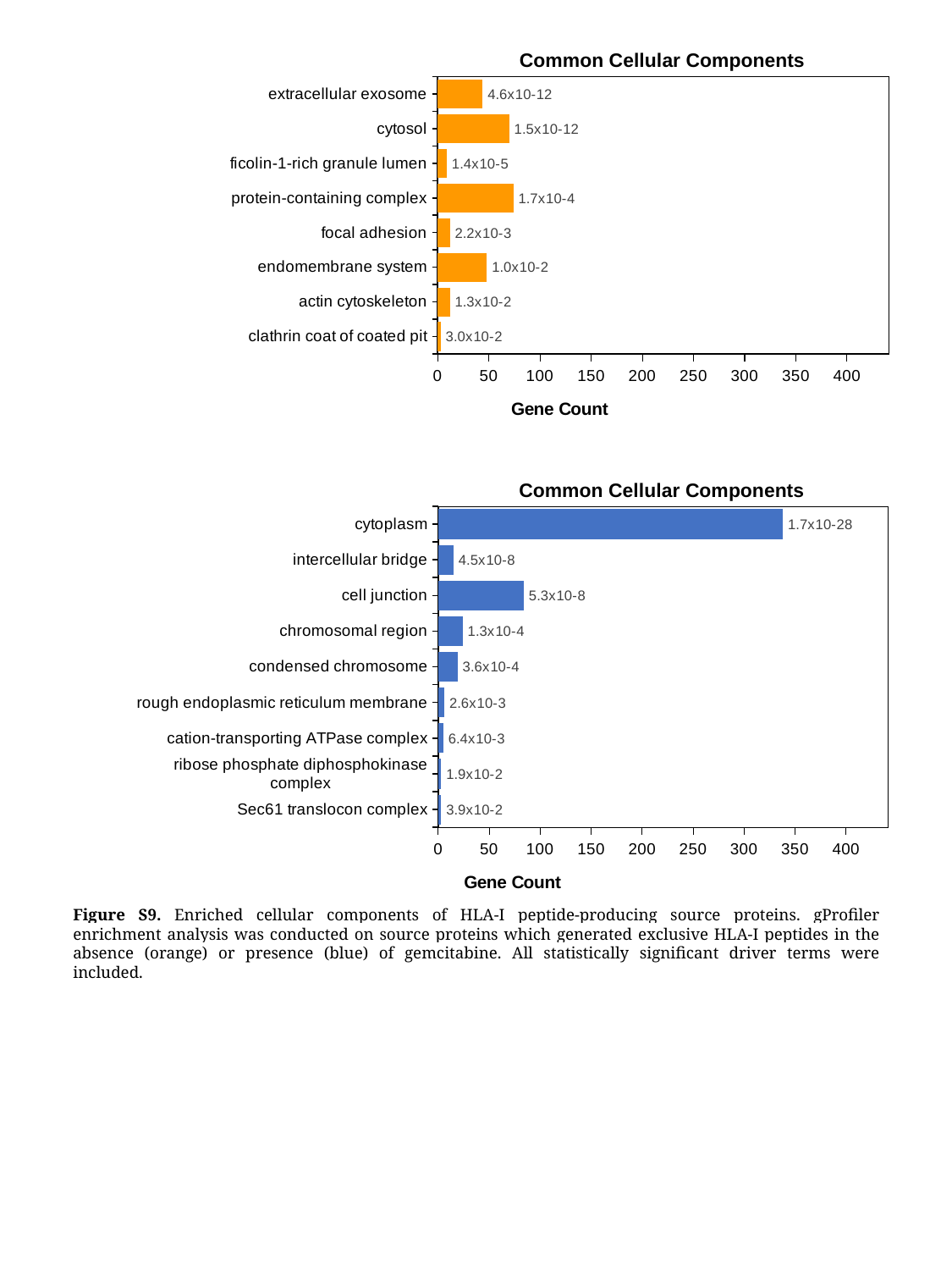

### Chart: Common Cellular Compartments
| Category | |
|---|---|
| extracellular exosome | 44.0 |
| cytosol | 70.0 |
| ficolin-1-rich granule lumen | 9.0 |
| protein-containing complex | 74.0 |
| focal adhesion | 12.0 |
| endomembrane system | 48.0 |
| actin cytoskeleton | 12.0 |
| clathrin coat of coated pit | 3.0 |Common Cellular Components
### Chart: Common Cellular Compartments
| Category | |
|---|---|
| cytoplasm | 338.0 |
| intercellular bridge | 15.0 |
| cell junction | 84.0 |
| chromosomal region | 24.0 |
| condensed chromosome | 19.0 |
| rough endoplasmic reticulum membrane | 6.0 |
| cation-transporting ATPase complex | 5.0 |
| ribose phosphate diphosphokinase complex | 3.0 |
| Sec61 translocon complex | 3.0 |Common Cellular Components
Figure S9. Enriched cellular components of HLA-I peptide-producing source proteins. gProfiler enrichment analysis was conducted on source proteins which generated exclusive HLA-I peptides in the absence (orange) or presence (blue) of gemcitabine. All statistically significant driver terms were included.
