# Supplementary material for: Gemcitabine Modulates HLA-I Regulation to Improve Tumor Antigen Presentation by Pancreatic Cancer Cells
Source: Int J Mol Sci. 2024 Mar 11;25(6):3211. doi: 10.3390/ijms25063211 (PMC10970070; doi:10.3390/ijms25063211)
Supplement: Supplementary file 1 [file ijms-25-03211-s001.zip › Table S1.pdf]

Table S1. Gemcitabine-exclusive HLA-I peptides mapped to a single source protein.

	Source protein	*Peptide*	*Assigned HLA-I allotype(s)*	*IC50 (nM)*	*Immunogenicity score(s)*[#]	*Accession number(s)*	*Repeated peptide sequence*
1	26S proteasome regulatory subunit 6B	LVIGQFLEA	A*02:06	17.5	0.124	P43686	
2	7-dehydrocholesterol reductase	GQWGRAWEV	A*02:06	2.7	0.439	Q9UBM7	
3	Adenylate kinase 2, mitochondrial	FSIPDSLLI	A*02:06	53.5	-0.118	P54819	
4	Adhesion G-protein coupled receptor G6	IQDPVIFYV	A*02:06	2.8	0.258	Q86SQ4	
5	ADP-ribosylation factor-like protein 6-interacting protein 1	YLDPSVLSGV	A*02:06	8.8	-0.255	Q15041	‡
6	Aminoacyl tRNA synthase complex-interacting multifunctional protein 2	TVADVVLWSV	A*02:06	5.8	0.195	Q13155	
7	Angio-associated migratory cell protein	SGLLKVWQV	A*02:06	229.7	-0.067	Q13685	
8	Anillin	FALVGSYTL	A*02:06	29.7	-0.065	Q9NQW6	
9	AP-3 complex subunit mu-2	FIPPDGNFRL	A*02:06	217.4	0.163	P53677	
			C*01:02	277.8	0.166		
10	Apoptosis inhibitor 5	RVADILTQL	A*02:06	13.3	0.119	Q9BZZ5	†
11	Aspartate--tRNA ligase, mitochondrial	GGLDRYFQV	A*02:06	437.7	0.097	Q6PI48	
12	ATPase GET3	FAMEIDPSL	A*02:06	6.6	0.088	O43681	
			C*01:02	368.2	0.145		

13	ATP-dependent DNA/RNA helicase DHX36	YQLPEILRT	A*02:06	31.1	0.229	Q9H2U1	
14	Bax inhibitor 1	NVFFGSIWL	A*02:06	151.3	0.275	P55061	
15	Biogenesis of lysosome-related organelles complex 1 subunit 1	TIATALEYV	A*02:06	7.2	0.162	P78537	
16	Calponin-2	HQLENLSNFI	A*02:06	43.6	-0.013	Q99439	
17	Carboxypeptidase D	SLITLIEKV	A*02:06	13.9	0.155	O75976	
18	Catenin alpha-1	KQDETQTKI	A*02:06	328.0	-0.057	P35221	
19	Caveolae-associated protein 1	SLLDKIIGA	A*02:06	14.7	0.066	Q6NZI2	†
20	CCAAT/enhancer-binding protein beta	RAIDFSPYL	A*02:06	11.9	0.012	P17676	
			C*01:02	401.4	-0.031		
			C*07:02	373.3	0.012		
21	CCN family member 1	RALALVVTL	A*02:06	91.8	0.121	O00622	
22	Centrosomal protein of 55 kDa	NQITQLESL	A*02:06	71.5	-0.053	Q53EZ4	
23	Cold shock domain-containing protein E1	GVIEKLLTS	A*02:06	271.8	-0.063	O75534	†
24	Cold-inducible RNA-binding protein	GQISEVVVV	A*02:06	16.0	0.072	Q14011	†
25	Conserved oligomeric Golgi complex subunit 4	SQMATILNL	A*02:06	5.9	0.132	Q9H9E3	
26	Cyclin-dependent kinase 12	AQLELISRL	A*02:06	10.3	0.102	Q9NYV4	
27	Cyclin-dependent kinase 13	AQLELISRI	A*02:06	9.6	0.102	Q14004	
28	Cytoplasmic dynein 1 heavy chain 1	IITKDLFQV	A*02:06	10.3	-0.162	Q14204	†
29	Cytoplasmic dynein 1 heavy chain 1	SVLDDNKLLTL	A*02:06	249.6	-0.173	Q14204	
30	Cytosolic 5'-nucleotidase 3A	YAIEVDPVL	A*02:06	34.2	0.220	Q9H0P0	

31	D-dopachrome decarboxylase	KIGTVMTFL	A*02:06	229.6	0.026	P30046	
32	Death-associated protein kinase 1	FLKQILNGV	A*02:06	15.9	-0.053	P53355	
33	Disks large homolog 5	SQLELGVHL	A*02:06	18.2	0.172	Q8TDM6	
34	DISP complex protein LRCH3	HLPEELAEL	A*02:06	31.9	0.276	Q96II8	
			C*01:02	58.8	0.279		
35	DNA mismatch repair protein Msh6	KIIGIMEEV	A*02:06	3.4	0.185	P52701	
36	DNA repair protein XRCC1	FQNPFRSEL	A*02:06	46.2	0.068	P18887	
			C*07:02	131.3	0.068		
37	DNA replication licensing factor MCM2	YVIEDDVNM	A*02:06	26.8	0.217	P49736	
38	DNA topoisomerase 3-alpha	FVPEIFHRI	A*02:06	5.3	0.394	Q13472	
			C*01:02	215.1	0.398		
39	Dolichyl-diphosphooligosaccharide--protein glycosyltransferase subunit 4	VQLAIFANM	A*02:06	47.5	0.305	P0C6T2	†
40	Dr1-associated corepressor	FLKDLVASV	A*02:06	9.2	-0.083	Q14919	
41	Dual specificity protein phosphatase 1	KLDEAFEFV	A*02:06	4.3	0.409	P28562	
42	E3 ubiquitin-protein ligase HUWE1	HVLDTLIQL	A*02:06	21.3	0.091	Q7Z6Z7	
43	E3 ubiquitin-protein ligase ZNF598	FQEEDFPAL	A*02:06	8.8	0.279	Q86UK7	
44	E3 ubiquitin-protein transferase MAEA	GVVEKLSVL	A*02:06	309.4	-0.222	Q7L5Y9	
45	Ectoderm-neural cortex protein 1	YLPELLQTV	A*02:06	3.2	0.001	O14682	†
			C*01:02	69.1	0.004		

46	EH domain-containing protein 1	TVLGKIWKL	A*02:06	26.3	0.007	Q9H4M9	
47	EKC/KEOPS complex subunit LAGE3	SVINFLDQL	A*02:06	11.1	0.091	Q14657	
48	EKC/KEOPS complex subunit TPRKB	IVDPFQILV	A*02:06	13.7	0.107	Q9Y3C4	
49	Elongation factor 1-gamma	FSWEGAFQHV	A*02:06	9.2	0.275	P26641	†
50	Epithelial cell adhesion molecule	YQLDPKFITSIL	A*02:06	75.5	0.016	P16422	
			B*07:02	204.9	0.016		
51	Estradiol 17-beta-dehydrogenase 11	ILPERFLAV	A*02:06	6.0	0.271	Q8NBQ5	
			C*01:02	35.7	0.275		
52	Eukaryotic initiation factor 4A-I	QIYDIFQKL	A*02:06	118.2	0.037	P60842	
53	Eukaryotic peptide chain release factor subunit 1	SVLGAITSV	A*02:06	4.9	0.130	P62495	
54	Eukaryotic translation initiation factor 3 subunit L	SVLNVLHSL	A*02:06	11.0	-0.050	Q9Y262	†
55	Eukaryotic translation initiation factor 3 subunit M	YIPTELDQV	A*02:06	20.2	0.074	Q7L2H7	†
56	Eukaryotic translation initiation factor 4 gamma 1	GVIDLIFEKA	A*02:06	33.7	0.253	Q04637	†
57	Eukaryotic translation initiation factor 4 gamma 2	AIISELVSI	A*02:06	9.7	-0.098	P78344	†
58	Exosome complex component RRP4	FLQEGDLISA	A*02:06	23.2	0.123	Q13868	‡
59	Exportin-2	AIIPYIPTL	A*02:06	6.1	0.167	P55060	
60	Fanconi anemia group B protein	AVWKESFQV	A*02:06	9.5	-0.172	Q8NB91	
61	Fatty acid synthase	QTPEFFQDV	A*02:06	119.8	0.237	P49327	
62	Fc receptor-like protein 1	LLSTLGPATV	A*02:06	272.9	0.053	Q96LA6	
63	Ferritin light chain	YLQASYTYL	A*02:06	12.5	-0.132	P02792	†

			C*01:02	350.1	-0.095		
64	Fibronectin type III domain-containing protein 3B	AQQVILVQV	A*02:06	23.6	0.090	Q53EP0	
65	Follicle-stimulating hormone receptor	LSQLYVMSLL	A*02:06	173.2	-0.324	P23945	
66	G2/mitotic-specific cyclin-B1	ILIDWLVQV	A*02:06	2.6	0.238	P14635	
67	G2/mitotic-specific cyclin-B2	ILVDWLVQV	A*02:06	3.3	0.208	O95067	
68	Glutamine and serine-rich protein 1	AQLPSLLSV	A*02:06	3.2	-0.292	Q2KHR3	
69	Glyceraldehyde-3-phosphate dehydrogenase	ILGYTEHQV	A*02:06	419.4	0.099	P04406	
70	Glycerol-3-phosphate acyltransferase 3	VLLPLRVTL	A*02:06	132.6	0.081	Q53EU6	
71	Glycogen phosphorylase, liver form	MIIKLITSV	A*02:06	4.6	-0.123	P06737	
72	Glycolipid transfer protein	YTQMNAELNYKV	A*02:06	219.9	-0.231	Q9NZD2	
73	Golgi-specific brefeldin A-resistance guanine nucleotide exchange factor 1	FVDPNGKISL	A*02:06	233.7	-0.165	Q92538	†
			C*01:02	262.2	-0.172		
74	GRIP and coiled-coil domain-containing protein 2	TQLELSEQL	A*02:06	42.0	-0.053	Q8IWJ2	
75	Growth arrest and DNA damage-inducible protein GADD45 alpha	RTITVGVYEA	A*02:06	247.3	0.250	P24522	
76	GTP-binding protein 4	QSLEYLEQV	A*02:06	25.0	0.100	Q9BZE4	
77	Histone deacetylase complex subunit SAP18	ATLKELTSL	A*02:06	92.5	-0.197	O00422	
78	Histone-lysine N-methyltransferase 2A	AVFPDDMPTL	A*02:06	84.2	-0.082	Q03164	†
79	Importin-9	TAIDILTTV	A*02:06	7.3	0.240	Q96P70	†
80	Interleukin enhancer-binding factor 2	STVKVLIRL	A*02:06	192.6	-0.031	Q12905	†

81	Interleukin-6 receptor subunit beta	KILDYEVTL	A*02:06	27.1	0.167	P40189	
82	Inverted formin-2	EVVERLLSV	A*02:06	48.1	0.048	Q27J81	
83	Keratin, type II cytoskeletal 80	NLLQVLEKV	A*02:06	30.31	-0.1319	Q6KB66	
84	KH homology domain-containing protein 4	VQDKLFVGL	A*02:06	75.5	-0.056	Q7Z7F0	
85	Kinesin-like protein KIF11	TVLQELINV	A*02:06	11.2	0.075	P52732	†
86	Kinesin-like protein KIF1C	GQVDMDIKL	A*02:06	82.9	-0.128	O43896	†
87	Kinesin-like protein KIF3B	KVVDVDVKL	A*02:06	105.3	0.006	O15066	
88	Laminin subunit alpha-3	SLLQTNIAL	A*02:06	103.4	0.047	Q16787	
89	Large ribosomal subunit protein mL65	SVPIEIPTI	A*02:06	235.2	0.366	Q9NP92	
			C*01:02	332.2	0.370		
90	Large ribosomal subunit protein uL30	DLIHEIYTV	A*02:06	135.3	0.318	P18124	
91	Large ribosomal subunit protein uL6	LILEGNDIEL	A*02:06	277.1	0.316	P32969	
92	Ligand-dependent nuclear receptor-interacting factor 1	SVLQSIEKI	A*02:06	391.1	-0.197	Q5T3J3	
93	Lipase member H	FCMYHYFVDI	A*02:06	367.2	0.125	Q8WWY8	
94	Lysine--tRNA ligase	KLVGEFLEV	A*02:06	3.9	0.304	Q15046	
95	Lysyl oxidase homolog 4	RIPEEGLLEV	A*02:06	61.8	0.266	Q96JB6	
96	Macrophage migration inhibitory factor	FLSELTQQL	A*02:06	7.2	-0.093	P14174	
			C*07:02	288.4	-0.093		
97	Matrin-3	YTIPDEYRI	A*02:06	29.0	0.175	P43243	
98	Mediator of RNA polymerase II transcription subunit 23	LIPKFLTEV	A*02:06	6.8	-0.026	Q9ULK4	

			C*01:02	275.7	-0.022		
99	Metaxin-3	SQIPFPLSL	A*02:06	13.9	0.030	Q5HYI7	
			C*07:02	337.5	0.030		
100	Microfibrillar-associated protein 1	TILPKVMQV	A*02:06	20.1	-0.402	P55081	†
101	Microtubule-actin cross-linking factor 1, isoforms 1/2/3/4/5	ALLDWVTSV	A*02:06	3.0	0.209	Q9UPN3	
102	Minor histocompatibility antigen H13	FGTNVMVTV	A*02:06	55.7	-0.061	Q8TCT9	
103	Mitochondrial carrier homolog 1	VQVDGKIGL	A*02:06	125.7	-0.002	Q9NZJ7	‡
104	Mitochondrial potassium channel	SVLGALIGV	A*02:06	3.0	0.190	Q96ER9	
105	Mitotic checkpoint serine/threonine-protein kinase BUB1 beta	KIVDFSYSV	A*02:06	2.5	-0.106	O60566	
106	Monocarboxylate transporter 1	YAFPKSITV	A*02:06	7.5	-0.204	P53985	
			C*01:02	282.5	-0.242		
			C*07:02	222.6	-0.204		
107	Myoglobin	GLSDGEWQL	A*02:06	52.5	0.215	P02144	
108	NADH dehydrogenase [ubiquinone] 1 alpha subcomplex subunit 13	LQIEDFEARI	A*02:06	32.6	0.437	Q9P0J0	
109	Nardilysin	TQVTALIKL	A*02:06	79.5	0.066	O43847	
110	NEDD4-binding protein 2	AAIEIFEKV	A*02:06	10.1	0.342	Q86UW6	
111	Nuclear factor NF-kappa-B p100 subunit	SVIEQIVYV	A*02:06	2.4	0.189	Q00653	‡
112	Nuclear pore complex protein Nup50	SVLGSLSSV	A*02:06	5.2	-0.377	Q9UKX7	
113	Nucleolar GTP-binding protein 2	IVLKGVVQV	A*02:06	16.9	-0.182	Q13823	

114	Nucleolar protein 56	VIAEILRGV	A*02:06	7.9	0.296	O00567	
115	Nucleolysin TIAR	SQWGQWSQV	A*02:06	8.5	-0.006	Q01085	
116	ORM1-like protein 2	ATYVFLHTV	A*02:06	6.3	0.194	Q53FV1	
117	PDZ domain-containing protein GIPC1	SVIDHIHLI	A*02:06	4.4	0.243	O14908	†
118	Pecanex-like protein 3	GVLENIFGV	A*02:06	2.4	0.335	Q9H6A9	
119	Peptidyl-prolyl cis-trans isomerase C	VTAKVFFDV	A*02:06	29.3	0.058	P45877	
120	PHD finger protein 20-like protein 1	NLLTHIENV	A*02:06	18.1	0.273	A8MW92	
121	Phospholipase A-2-activating protein	SLISTILEV	A*02:06	5.9	0.089	Q9Y263	
122	Plastin-1	EIVEKILSV	A*02:06	81.3	-0.077	Q14651	
123	Plectin	YVIDPIKGLKL	A*02:06	385.4	-0.129	Q15149	
124	Pogo transposable element with ZNF domain	NQVGIVLNV	A*02:06	10.4	0.203	Q7Z3K3	
125	Probable ATP-dependent RNA helicase DDX52	FQMPTPIQM	A*02:06	9.1	0.004	Q9Y2R4	
			C*07:02	98.3	0.004		
126	Proteasome maturation protein	QQVQRLPFL	A*02:06	31.3	-0.004	Q9Y244	
127	Protein cereblon	QVIPVLPQV	A*02:06	5.1	-0.015	Q96SW2	
128	Protein disulfide-isomerase A3	LAAARLAAA	A*02:06	201.5	0.148	P30101	
129	Protein FAM171B	VLLKARLVPA	A*02:06	78.4	-0.114	Q6P995	
130	Protein FAM83D	TQTAVITRI	A*02:06	206.1	0.280	Q9H4H8	
131	Protein flightless-1 homolog	SLFPGKLEV	A*02:06	12.4	-0.094	Q13045	†
132	Protein flightless-1 homolog	SQVEIKLSL	A*02:06	42.8	-0.065	Q13045	
133	Protein mono-ADP-ribosyltransferase PARP4	FIFSDTHEL	A*02:06	4.2	0.015	Q9UKK3	

			C*01:02	459.2	-0.023		
			C*07:02	245.4	0.015		
134	Protein PRRC2C	SQFQQEFPSL	A*02:06	84.0	-0.090	Q9Y520	
135	Protein RRP5 homolog	VQYNEPLKV	A*02:06	61.7	-0.056	Q14690	
136	Protein S100-A11	GQLDFSEFLNL	A*02:06	112.8	0.166	P31949	
137	Protein transport protein Sec24A	FLLHIQQQV	A*02:06	7.3	-0.116	O95486	
138	Protein unc-45 homolog A	NLLDLLTEV	A*02:06	4.1	0.089	Q9H3U1	
139	Protein unc-80 homolog	RQTSQVETV	A*02:06	64.0	-0.121	Q8N2C7	
140	Ras association domain-containing protein 1	GSYTGFIKV	A*02:06	62.5	0.167	Q9NS23	
141	Ras GTPase-activating protein-binding protein 1	YQDEVFGGF	A*02:06	53.8	0.307	Q13283	
142	Ras-related protein Rab-18	ATIGVDFKV	A*02:06	14.0	0.111	Q9NP72	
143	Renin receptor	NAVVELVTV	A*02:06	193.1	0.200	O75787	
144	Replication protein A 32 kDa subunit	TLVDEVFRI	A*02:06	19.9	0.301	P15927	
145	Reticulon-4	AQIDHYLGL	A*02:06	6.1	0.104	Q9NQC3	
146	Retinaldehyde dehydrogenase 3	KQFDKILEL	A*02:06	5.0	0.025	P47895	
			C*07:02	304.9	0.025		
147	Rhomboid domain-containing protein 2	YSIDLSERV	A*02:06	19.7	0.014	Q6NTF9	†
148	Rho-related GTP-binding protein RhoD	AVFQEAAEV	A*02:06	40.8	0.147	O00212	†
149	Ribonucleoside-diphosphate reductase large subunit	FVDPAQITM	A*02:06	36.3	0.060	P23921	†
150	Ribosomal L1 domain-containing protein 1	HIIENIVAV	A*02:06	4.3	0.321	O76021	

151	RING finger protein 214	ALFPAPLAQI	A*02:06	100.7	0.009	Q8ND24	
152	Serine palmitoyltransferase 1	STIKEVAQA	A*02:06	148.7	-0.072	O15269	
153	Serine/threonine-protein kinase DCLK1	SAIKYLHSL	A*02:06	28.6	-0.257	O15075	
154	Serine/threonine-protein phosphatase 4 catalytic subunit	AILELDEHL	A*02:06	130.1	0.211	P60510	
155	Serine/threonine-protein phosphatase 4 catalytic subunit	FVDRGFYSV	A*02:06	2.8	0.103	P60510	†
156	Small integral membrane protein 30	SVSTQLSLV	A*02:06	98.4	-0.284	A4D0T7	
157	Small ribosomal subunit protein eS19	KVPEWVDTV	A*02:06	7.5	0.393	P39019	
158	Small ribosomal subunit protein uS3	FVADGIFKA	A*02:06	3.6	0.166	P23396	†
159	Small ribosomal subunit protein uS9	KLLEPVLLL	A*02:06	12.2	0.109	P62249	
160	Speriolin-like protein	YVFPGVTRL	A*02:06	10.7	0.162	Q9H0A9	†
			C*07:02	229.8	0.162		
161	Splicing factor 3B subunit 3	GQWASVIRV	A*02:06	6.7	0.132	Q15393	†
162	Structural maintenance of chromosomes protein 5	KMIEDLQNEL	A*02:06	21.2	0.099	Q8IY18	
163	T-complex protein 1 subunit theta	EVISKLYAV	A*02:06	14.4	-0.324	P50990	‡
164	Threonine--tRNA ligase 1, cytoplasmic	TVIAKVNNV	A*02:06	31.4	-0.098	P26639	
165	Transcription elongation factor SPT5	VTTDIQVKV	A*02:06	249.7	-0.036	O00267	
166	Transcription factor Sp1	QVIPQFQTV	A*02:06	16.7	-0.046	P08047	
167	Transcriptional repressor protein YY1	YIEQTLVTV	A*02:06	36.9	0.001	P25490	
168	Transmembrane emp24 domain-containing protein 2	KMGLIFEV	A*02:06	60.0	0.324	Q15363	

169	Transmembrane emp24 domain-containing protein 9	SLFAGGMLRV	A*02:06	27.5	-0.001	Q9BVK6
170	Transmembrane protein 41B	FAIPGSIFL	A*02:06	11.4	0.090	Q5BJD5
			C*01:02	268.3	0.047	
			C*07:02	428.7	0.090	
171	Tripartite motif-containing protein 5	NIVEKLREV	A*02:06	52.4	-0.004	Q9C035
172	Tyrosine-protein kinase receptor UFO	NQDDSILKV	A*02:06	98.6	-0.142	P30530
173	Ubiquitin-like modifier-activating enzyme 6	ALLNILSEV	A*02:06	3.9	0.028	A0AVT1
174	UDP-N-acetylhexosamine pyrophosphorylase	NIANHFFTV	A*02:06	4.2	0.269	Q16222
175	UMP-CMP kinase	KIVPVEITI	A*02:06	187.7	0.272	P30085
176	Utrophin	NQWDGTQHGV	A*02:06	45.1	0.103	P46939
177	Voltage-dependent anion-selective channel protein 2	YQLDPTASISA	A*02:06	120.7	-0.056	P45880
178	V-type proton ATPase subunit d 1	RLYPEGLAQL	A*02:06	44.6	0.073	P61421
			A*24:02	390.3	0.073	
			C*01:02	403.9	0.074	
179	Zinc finger protein 217	ATINEVVQV	A*02:06	16.1	0.140	O75362
180	26S proteasome regulatory subunit 8	YYLSKIEEL	A*24:02	26.8	-0.112	P62195
			C*07:02	30.8	-0.112	
181	Abnormal spindle-like microcephaly-associated protein	SYNPLWLRI	A*24:02	14.4	0.205	Q8IZT6
182	Activated CDC42 kinase 1	QYLKVEQLF	A*24:02	13.3	-0.190	Q07912
183	AF4/FMR2 family member 4	SYVQVTSNF	A*24:02	12.2	-0.170	Q9UHB7

			C*07:02	185.8	-0.170		
184	AKT-interacting protein	LYQDGVFKF	A*24:02	9.7	0.029	Q9H8T0	
185	Alpha-enolase	RYISPDQLADL	A*24:02	460.5	-0.190	P06733	†
186	AP-2 complex subunit sigma	AYLEAIHNF	A*24:02	9.0	0.284	P53680	
			C*07:02	283.5	0.284		
187	Aquaporin-3	LYYDAIWHF	A*24:02	13.6	0.390	Q92482	
			C*07:02	124.0	0.390		
188	Aspartate aminotransferase, mitochondrial	EYLPIGGLAEF	A*24:02	250.5	0.262	P00505	‡
189	ATPase family AAA domain-containing protein 2	LYPEVFEKF	A*24:02	7.9	0.206	Q6PL18	†
			C*07:02	237.1	0.206		
190	BLOC-2 complex member HPS3	IYPWVHVVI	A*24:02	19.6	0.349	Q969F9	
			C*07:02	405.0	0.349		
191	Brain-specific angiogenesis inhibitor 1-associated protein 2-like protein 2	IYKSIMEQF	A*24:02	15.2	-0.255	Q6UXY1	†
			C*07:02	195.5	-0.255		
192	Breast cancer anti-estrogen resistance protein 3	AYSRVQYQF	A*24:02	10.4	-0.141	O75815	†
			C*07:02	380.8	-0.141		
193	Carnitine O-palmitoyltransferase 1, liver isoform	VYPASPSSWLI	A*24:02	27.6	-0.273	P50416	
194	CCAAT/enhancer-binding protein zeta	LYQHEINLF	A*24:02	11.1	0.206	Q03701	
195	Cellular tumor antigen p53	TYSPALNKMF	A*24:02	46.3	-0.328	P04637	†
196	Cip1-interacting zinc finger protein	RYFKTPRKF	A*24:02	30.6	-0.234	Q9ULV3	

			C*07:02	149.3	-0.234		
197	Cleavage stimulation factor subunit 3	AYIDYLSHL	A*24:02	63.5	-0.069	Q12996	
			C*07:02	74.0	-0.069		
198	Condensin-2 complex subunit H2	LFIATSQKF	A*24:02	39.0	-0.259	Q6IBW4	
199	Cytochrome b-245 light chain	AYSIVAGVF	A*24:02	86.8	0.210	P13498	†
200	Cytoplasmic dynein 1 heavy chain 1	RYKLYQEMF	A*24:02	26.2	-0.212	Q14204	‡
			C*07:02	496.6	-0.212		
201	Cytoplasmic dynein 2 intermediate chain 2	RWSPVRPLVF	A*24:02	16.5	0.030	Q96EX3	‡
202	Cytoplasmic FMR1-interacting protein 1	LYQPNFNTNF	A*24:02	8.8	0.082	Q7L576	
			C*07:02	408.7	0.082		
203	Deoxyribonuclease TATDN1	KYFEKQFEL	A*24:02	46.0	-0.023	Q6P1N9	
			C*07:02	67.4	-0.023		
204	DNA ligase 1	KYPDIISRI	A*24:02	24.3	0.164	P18858	†
			C*07:02	425.3	0.164		
205	DNA repair protein RAD50	EYVEKFYRI	A*24:02	30.0	0.041	Q92878	
206	DNA topoisomerase 3-beta-1	KYLQSTISF	A*24:02	12.0	-0.229	O95985	
			C*07:02	299.2	-0.229		
207	DNA-(apurinic or apyrimidinic site) endonuclease	YFLLSHSLL	A*24:02	217.3	-0.292	P27695	
			C*07:02	374.3	-0.292		
208	DNA-dependent protein kinase catalytic subunit	YYIQNGIQSF	A*24:02	8.1	-0.116	P78527	
			C*07:02	73.0	-0.116		

209	DNA-directed RNA polymerase I subunit RPA1	VYQLRFQFL	A*24:02	21.8	0.082	O95602	
			C*07:02	172.4	0.082		
210	E3 ubiquitin-protein ligase ZNF598	DYAYLREHF	A*24:02	291.9	0.150	Q86UK7	
211	EH domain-containing protein 2	KYDEIFYNL	A*24:02	52.5	0.341	Q9NZN4	†
			C*07:02	422.8	0.341		
212	Elongation of very long chain fatty acids protein 1	QYPVIIHLI	A*24:02	11.2	0.314	Q9BW60	‡
213	Elongation of very long chain fatty acids protein 5	KYITQGQLLQF	A*24:02	52.9	-0.198	Q9NYP7	†
214	Epidermal growth factor receptor kinase substrate 8-like protein 1	EYTDVLQKI	A*24:02	299.5	-0.159	Q8TE68	
215	Ethanolamine kinase 1	AYLEAYKEF	A*24:02	32.2	0.008	Q9HBU6	
			C*07:02	298.8	0.008		
216	Eukaryotic initiation factor 4A-I	RYLSPKYIKMF	A*24:02	47.1	-0.554	P60842	
217	Eukaryotic translation initiation factor 2D	SYPDLLPTF	A*24:02	11.5	0.011	P41214	
			C*07:02	77.8	0.011		
218	Fanconi anemia group D2 protein	SYPKIIEEF	A*24:02	10.3	0.177	Q9BXW9	
			C*07:02	133.6	0.177		
219	Fanconi anemia group I protein	FYQPKIQQF	A*24:02	12.6	-0.299	Q9NVI1	†
			C*07:02	18.2	-0.299		
220	Filamin-B	YYPSTPGRYSI	A*24:02	93.1	-0.161	O75369	
221	G1/S-specific cyclin-D3	RYVPRASYF	A*24:02	12.1	-0.052	P30281	
			C*07:02	28.9	-0.052		

222	Glutathione peroxidase 3	QYAGKYVLF	A*24:02	11.1	-0.138	P22352	
			C*07:02	350.0	-0.138		
223	Guanine nucleotide-binding protein subunit alpha-15	RYLDEINLL	A*24:02	24.8	0.230	P30679	
			C*07:02	256.0	0.230		
224	Heat shock protein beta-1	RLPEEWSQW	A*24:02	193.1	0.196	P04792	†
225	Helicase-like transcription factor	SYPTFFPRF	A*24:02	5.8	0.281	Q14527	
			C*07:02	65.5	0.281		
226	Histone-lysine N-methyltransferase ASH1L	TYDYNFHSF	A*24:02	17.8	0.038	Q9NR48	
			C*07:02	59.6	0.038		
227	Importin-7	YYNPHLLLNTL	A*24:02	143.4	0.003	O95373	
			C*07:02	151.7	0.003		
228	Integrin beta-4	DYTIGFGKF	A*24:02	487.3	0.192	P16144	†
229	Iron-responsive element-binding protein 2	TYLKAVKLF	A*24:02	11.1	-0.332	P48200	†
			C*07:02	413.5	-0.332		
230	Kinesin-like protein KIF2C	IYNGKLFDL	A*24:02	161.9	-0.077	Q99661	†
			C*07:02	237.7	-0.077		
231	La-related protein 4	VYSIVPQSW	A*24:02	36.4	-0.084	Q71RC2	†
232	Large ribosomal subunit protein eL19	LYLKVKGNVF	A*24:02	74.0	-0.340	P84098	†
233	Large ribosomal subunit protein eL27	KWFFQKLRF	A*24:02	32.3	-0.139	P61353	
234	Large ribosomal subunit protein uL22	RWPKKSAEFLL	A*24:02	232.8	-0.367	P18621	

235	Leucine-rich PPR motif-containing protein, mitochondrial	IYKPVTDFF	A*24:02	9.1	0.083	P42704	‡
			C*07:02	84.4	0.083		
236	Melanophilin	YYEHVKARF	A*24:02	62.0	-0.034	Q9BV36	
			C*07:02	238.8	-0.034		
237	Mitofusin-2	AYIQESATF	A*24:02	18.8	-0.076	O95140	
			C*07:02	344.7	-0.076		
238	Molybdenum cofactor sulfurase	VYLDHAGATLF	A*24:02	28.4	0.184	Q96EN8	
239	Myotubularin-related protein 12	LYIPIFSTF	A*24:02	4.6	0.155	Q9C0I1	
			C*07:02	84.1	0.155		
240	Myotubularin-related protein 6	NYSNIRFQF	A*24:02	12.1	0.149	Q9Y217	
			C*07:02	71.9	0.149		
241	N-acetyllactosaminide beta-1,3-N-acetylglucosaminyltransferase 2	NYRDTFFNL	A*24:02	104.9	0.282	Q9NY97	
			C*07:02	109.0	0.282		
242	NADH dehydrogenase [ubiquinone] 1 beta subcomplex subunit 6	VYKKSIFVF	A*24:02	9.2	-0.200	O95139	
			C*07:02	104.6	-0.200		
243	Neutral cholesterol ester hydrolase 1	KYFLKPEVL	A*24:02	143.9	-0.085	Q6PIU2	
			C*07:02	340.3	-0.085		
244	Nuclear autoantigenic sperm protein	NYVQAVEEF	A*24:02	37.5	0.117	P49321	
			C*07:02	221.8	0.117		

245	Nuclear pore complex protein Nup107	AYLEAHETF	A*24:02	11.6	0.273	P57740	
			C*07:02	292.5	0.273		
246	Nuclear receptor corepressor 1	TYIPGTQITF	A*24:02	13.0	0.129	O75376	
			C*07:02	309.5	0.129		
247	Opsin-3	VYNPVIYVF	A*24:02	5.3	0.173	Q9H1Y3	
			C*07:02	33.3	0.173		
248	ORM1-like protein 2	VLLPKLPQF	A*24:02	481.0	-0.312	Q53FV1	
249	Oxysterol-binding protein-related protein 2	EYMEHVYLI	A*24:02	6.9	0.105	Q9H1P3	
			C*07:02	483.4	0.105		
250	Paired amphipathic helix protein Sin3a	VYAQVARLF	A*24:02	8.3	0.010	Q96ST3	
			C*07:02	78.9	0.010		
251	Paired amphipathic helix protein Sin3b	VFTEVANLF	A*24:02	33.1	0.178	O75182	
252	Partitioning defective 3 homolog B	VYFPDQHINF	A*24:02	17.7	0.075	Q8TEW8	†
			C*07:02	395.0	0.075		
253	Phospholipase A-2-activating protein	IYFPKKEAVTF	A*24:02	48.7	-0.201	Q9Y263	
254	Plectin	IYLEKLKTI	A*24:02	30.5	-0.283	Q15149	
255	Polycystin family receptor for egg jelly	SYILTHFIF	A*24:02	10.4	0.277	Q9NTG1	
			C*07:02	442.6	0.277		
256	Pre-mRNA-processing-splicing factor 8	NYLHLDYNF	A*24:02	8.5	0.032	Q6P2Q9	
257	Probable C-mannosyltransferase DPY19L4	NYSPYVNYF	A*24:02	13.5	-0.037	Q7Z388	†

			C*07:02	26.2	-0.037		
258	Probable dolichyl pyrophosphate Glc1Man9GlcNAc2 alpha-1,3-glucosyltransferase	HYSLFPLLF	A*24:02	9.6	0.023	Q9BVK2	
			C*07:02	122.5	0.023		
259	Programmed cell death protein 6	KYITDWQNVF	A*24:02	11.4	0.229	O75340	†
260	Prohibitin 1	TYLPAGQSVLL	A*24:02	183.8	-0.184	P35232	
261	Prostaglandin E synthase 3	KWYDRRDYVF	A*24:02	30.3	0.164	Q15185	
262	Proteasome subunit beta type-7	VYAPPVGGFSF	A*24:02	19.7	0.098	Q99436	
263	Protein AATF (Apoptosis-antagonizing transcription factor)	PQPDVFPLF	A*24:02	257.4	0.153	Q9NY61	†
264	Protein CIP2A	TYGKIDLGF	A*24:02	27.5	-0.045	Q8TCG1	
265	Protein FAM104A	LYFHINQTL	A*24:02	45.7	0.119	Q969W3	
			C*07:02	83.2	0.119		
266	Protein mono-ADP-ribosyltransferase PARP3	FYTVIPHNF	A*24:02	10.8	0.197	Q9Y6F1	
			C*07:02	54.3	0.197		
267	Protein Niban 1	VYEEILHQI	A*24:02	47.8	0.212	Q9BZQ8	†
268	Protein PPP4R3C	AYIPKLLQL	A*24:02	97.2	-0.265	Q6ZMV5	
			C*07:02	118.5	-0.265		
269	Protein SGT1 homolog	NYAAALETF	A*24:02	10.3	0.187	Q9Y2Z0	
			C*07:02	214.5	0.187		
270	Protein unc-119 homolog B	IYSIDFTRF	A*24:02	15.9	0.275	A6NIH7	
			C*07:02	432.8	0.275		

271	Protein zwilch homolog	FYSRLLQKF	A*24:02	13.1	-0.247	Q9H900	
			C*07:02	102.0	-0.247		
272	Pterin-4-alpha-carbinolamine dehydratase	VYNKVHITL	A*24:02	25.4	-0.013	P61457	
			C*07:02	74.5	-0.013		
273	Retinoic acid-induced protein 3	LYAPYSTHF	A*24:02	9.4	-0.106	Q8NFJ5	
			C*07:02	38.6	-0.106		
274	Selenoprotein N	SYLPFTEAF	A*24:02	9.0	0.243	Q9NZV5	
			C*07:02	59.9	0.243		
275	Semaphorin-7A	AYDDKIYYF	A*24:02	126.2	-0.060	O75326	
			C*07:02	200.9	-0.060		
276	Separin	LYSNIAFNL	A*24:02	30.3	0.201	Q14674	
			C*07:02	223.9	0.201		
277	Serine--tRNA ligase, cytoplasmic	YFLKGVLVF	A*24:02	51.4	-0.134	P49591	
278	SET and MYND domain-containing protein 4	NYNAVFNLL	A*24:02	30.8	0.176	Q8IYR2	
			C*07:02	96.3	0.176		
279	Short transient receptor potential channel 4-associated protein	SYADQMFLL	A*24:02	33.5	-0.151	Q8TEL6	
			C*07:02	62.4	-0.151		
280	Signal peptidase complex subunit 2	KYVENFGLI	A*24:02	12.3	0.240	Q15005	
281	Small ribosomal subunit protein uS8	LLPSRQFGF	A*24:02	306.8	-0.110	P62244	
282	Sodium bicarbonate cotransporter 3	VYIGVHVPF	A*24:02	14.8	0.176	Q9Y6M7	†

			C*07:02	206.4	0.176		
283	Splicing factor 3B subunit 3	NYISGIQTI	A*24:02	12.8	-0.040	Q15393	
			C*07:02	214.7	-0.040		
284	StAR-related lipid transfer protein 7, mitochondrial	TYTDVTPRQFF	A*24:02	105.3	0.121	Q9NQZ5	†
285	Terminal nucleotidyltransferase 4A	LYGRNFNYL	A*24:02	33.1	0.159	Q5XG87	†
			C*07:02	370.5	0.159		
286	Terminal uridylyltransferase 4	VYEYVVERF	A*24:02	34.5	0.223	Q5TAX3	
287	Thimet oligopeptidase	KYPHYFPLL	A*24:02	7.8	0.120	P52888	
			C*07:02	117.5	0.120		
288	Thioredoxin-like protein 4B	SYIPSTVFF	A*24:02	9.3	0.011	Q9NX01	
			C*07:02	38.7	0.011		
289	Trafficking protein particle complex subunit 1	SYVRSLPFF	A*24:02	7.9	-0.047	Q9Y5R8	
			C*07:02	244.7	-0.047		
290	Transcription elongation factor SPT5	RYSRKGFLF	A*24:02	5.6	-0.087	O00267	
			C*07:02	219.7	-0.087		
291	tRNA-splicing endonuclease subunit Sen34	VYPGDPLRF	A*24:02	47.5	0.063	Q9BSV6	†
			C*07:02	400.6	0.063		
292	Tumor necrosis factor receptor superfamily member 21	VYSHLPDLL	A*24:02	66.7	-0.030	O75509	†
293	Tyrosine-protein kinase BAZ1B	RYQDIIHSI	A*24:02	6.3	0.170	Q9UIG0	
			C*07:02	166.4	0.170		
294	Tyrosine-protein kinase receptor UFO	VYTSKSDVWSF	A*24:02	56.0	-0.374	P30530	

295	U5 small nuclear ribonucleoprotein 200 kDa helicase	VYMDWYEKF	A*24:02	2.9	0.136	O75643	
			C*07:02	87.7	0.136		
296	Ubiquitin-like FUBI-ribosomal protein eS30 fusion protein	RFVNVVPTF	A*24:02	17.9	0.099	P62861	†
297	Ubiquitin-like modifier-activating enzyme 1	SYKNGFLNL	A*24:02	63.3	0.054	P22314	
			C*07:02	134.2	0.054		
298	Unconventional myosin-Ic	VYSRTFTWL	A*24:02	34.7	0.309	O00159	
			C*07:02	275.1	0.309		
299	Vacuolar protein sorting-associated protein 37B	KYQELQVLF	A*24:02	7.0	-0.028	Q9H9H4	
			C*07:02	298.4	-0.028		
300	V-type proton ATPase subunit d 1	AYLESFYKF	A*24:02	4.6	-0.083	P61421	
			C*07:02	424.5	-0.083		
301	Zinc phosphodiesterase ELAC protein 2	RYQQWMERF	A*24:02	5.6	0.011	Q9BQ52	
			C*07:02	454.8	0.011		
302	26S proteasome non-ATPase regulatory subunit 12	RPKDPNNLL	B*07:02	13.6	-0.077	O00232	
303	Cyclin-G-associated kinase	QPYGGFLDI	B*07:02	213.9	0.180	O14976	
304	Cysteine desulfurase	GPKGVGAIYI	B*07:02	464.1	0.184	Q9Y697	
305	DNA replication licensing factor MCM6	LPRGSIPRSL	B*07:02	6.6	-0.044	Q14566	
306	DNA replication licensing factor MCM7	RPADVIFATV	B*07:02	63.0	0.371	P33993	
307	Heat shock protein beta-1	MPKLATQSNEI	B*07:02	245.6	-0.221	P04792	
308	Integrin beta-4	GPRPSPWARL	B*07:02	15.7	0.106	P16144	

309	Large ribosomal subunit protein uL23	RPDGEKKAYVRL	B*07:02	355.0	-0.182	P62750	
310	Lipid droplet-regulating VLDL assembly factor AUP1	LPHVPLGVI	B*07:02	43.2	0.084	Q9Y679	
311	Maestro heat-like repeat-containing protein family member 6	APRPARPPP	B*07:02	41.0	0.077	A6NGR9	
312	Neuroplastin	LPSALALSLL	B*07:02	67.4	-0.144	Q9Y639	
313	Poly(rC)-binding protein 2	RPKPSSSPVIF	B*07:02	17.8	-0.462	Q15366	
314	Proteasome subunit alpha type-1	RPYGVGLLI	B*07:02	35.9	0.089	P25786	†
			B*59:01	221.0	0.089		
315	SEC14-like protein 1	APHEILIQI	B*07:02	461.4	0.275	Q92503	
316	Serine protease HTRA1	IPRAALLPLL	B*07:02	5.4	0.057	Q92743	
317	SH2 domain-containing protein 3A	RPRPGRPTA	B*07:02	5.0	0.101	Q9BRG2	
318	Solute carrier family 25 member 3	QPRRPRNLAA	B*07:02	22.8	0.116	Q00325	
319	Trafficking protein particle complex subunit 4	RPRLTSNEKL	B*07:02	3.4	-0.165	Q9Y296	
320	Transcription elongation factor SPT4	LPQGIVREL	B*07:02	36.2	0.267	P63272	
321	Transmembrane protein 214	GPRLPWTRL	B*07:02	8.9	0.266	Q6NUQ4	
322	Tricarboxylate transport protein, mitochondrial	APRAPRALAAA	B*07:02	21.4	0.179	P53007	
323	Zinc finger protein 526	APRLPITGL	B*07:02	5.7	0.173	Q8TF50	
324	Zinc finger protein 843	GLPAVPAVPL	B*07:02	91.5	0.130	Q8N446	
325	F-box only protein 33	LPSELIVHI	B*59:01	198.4	0.215	Q7Z6M2	
326	FIGNL1-interacting regulator of recombination and mitosis	LPSSVLHII	B*59:01	127.8	-0.085	Q9NSG2	
			B*07:02	210.2	-0.085		

327	Galectin-3	FPFESGKPFKI	B*59:01	367.7	-0.237	P17931
328	Lathosterol oxidase	LPWISILTV	B*59:01	110.7	0.183	O75845
329	Leucine--tRNA ligase, cytoplasmic	LPFEPVPVI	B*59:01	231.4	0.182	Q9P2J5
330	Vacuolar protein sorting-associated protein 26C	IPFEFPLHL	B*59:01	250.5	0.252	O14972
331	HLA class I histocompatibility antigen, A alpha chain	VMAPRTLLL	C*01:02	169.5	0.060	P04439
			A*02:06	205.9	0.073	
			A*24:02	297.7	0.073	
			C*07:02	232.9	0.073	
332	Threonylcarbamoyl-AMP synthase	YGLLPSHASYL	C*01:02	112.9	-0.257	Q86U90
333	5'-nucleotidase domain-containing protein 1	YYPNPPLVL	C*07:02	20.6	-0.017	Q5TFE4
			C*01:02	167.9	-0.013	
334	Cadherin-like and PC-esterase domain-containing protein 1	IYREDRPSL	C*07:02	304.4	0.082	A4D0V7
335	Cyclin-dependent kinases regulatory subunit 2	KYFDEHYEY	C*07:02	73.8	0.244	P33552
			A*24:02	462.1	0.244	
336	CYFIP-related Rac1 interactor B	IYNQVNVVL	C*07:02	85.9	-0.026	Q9NUQ9
			A*24:02	136.5	-0.026	
337	Elongin-A	NYRPLPSLEL	C*07:02	441.2	-0.123	Q14241
338	Microtubule-associated protein 1S	LYDELRNLL	C*07:02	200.4	0.134	Q66K74
339	Probable phospholipid-transporting ATPase IIB	YYEHEFVEL	C*07:02	118.4	0.366	O43861
			A*24:02	366.8	0.366	

340	Protein ITPRID2	LYENTFQEL	C*07:02	145.0	0.135	P28290
			A*24:02	280.0	0.135	
341	Protein transport protein Sec61 subunit alpha isoform 1	QYNTYPIKL	C*07:02	412.0	0.009	P61619
342	Structural maintenance of chromosomes protein 4	HYKPTPLYF	C*07:02	33.1	-0.065	Q9NTJ3
			A*24:02	41.0	-0.065	
343	V-type proton ATPase subunit d 1	LYPEGLAQL	C*07:02	180.0	0.085	P61421
			A*24:02	211.1	0.085	

[#] immunogenicity scores were calculated via the IEDB immunogenicity tool and allele-specific anchor residues were masked. Positive scores indicate more immunogenic peptides, whereas negative scores designate less immunogenic peptides

† peptide sequence identified in two of three mass spectrometry replicates

‡ peptide sequence identified in three of three mass spectrometry replicates
