# Supplementary material for: Gemcitabine Modulates HLA-I Regulation to Improve Tumor Antigen Presentation by Pancreatic Cancer Cells
Source: Int J Mol Sci. 2024 Mar 11;25(6):3211. doi: 10.3390/ijms25063211 (PMC10970070; doi:10.3390/ijms25063211)
Supplement: Supplementary file 1 [file ijms-25-03211-s001.zip › Table S2.pdf]

Table S2. Gemcitabine-exclusive HLA-I peptides mapped to multiple source proteins.

	Source Proteins	*Peptide*	*Assigned HLA-I allotype(s)*	*IC50 (nM)*	*Immunogenicity score(s)[#]*	*Accession numbers*	*Repeated peptide sequence*
1	Putative high mobility group protein B1-like 1, High mobility group protein B1, High mobility group protein B2	APKRPPSAF	B*07:02	11.3	-0.156	B2RPK0<br>P09429<br>P26583	
2	Heterogeneous nuclear ribonucleoprotein C-like 2, Heterogeneous nuclear ribonucleoprotein C-like 4, Heterogeneous nuclear ribonucleoprotein C-like 3, Heterogeneous nuclear ribonucleoprotein C-like 1, Heterogeneous nuclear ribonucleoproteins C1/C2	RVFIGNLNTL	A*02:06	157.1	0.205	B2RXH8<br>P0DMR1<br>B7ZW38<br>O60812<br>P07910	†
3	Protein transport protein Sec24A, Protein transport protein Sec24B	TYINPFVSF	A*24:02	10.5	0.074	O95486<br>O95487	
			C*07:02	101.1	0.074		
4	Mitogen-activated protein kinase kinase kinase kinase 4, Misshapen-like kinase 1, TRAF2 and NCK-interacting protein kinase	AWAPKPYHKF	A*24:02	80.8	-0.321	O95819<br>Q8N4C8<br>Q9UKE5	
5	Ferritin heavy chain, Ferritin, mitochondrial	RQINLELYA	A*02:06	50.4	0.109	P02794<br>Q8N4E7	
6	Guanine nucleotide-binding protein G(i) subunit alpha-2, Guanine nucleotide-binding protein G(z) subunit alpha	YYLNDLERI	A*24:02	22.1	0.116	P04899<br>P19086	†

7	Sodium/potassium-transporting ATPase subunit alpha-1, Sodium/potassium-transporting ATPase subunit alpha-2, Sodium/potassium-transporting ATPase subunit alpha-3, Potassium-transporting ATPase alpha chain 2, Sodium/potassium-transporting ATPase subunit alpha-4	NVPEGLLATV	A*02:06	86.2	0.165	P05023 P50993 P13637 P54707 Q13733	†
8	Alpha-enolase, Gamma-enolase, Beta-enolase	FIADLVVGL	A*02:06	4.0	0.118	P06733 P09104 P13929	
9	Tubulin beta chain, Tubulin beta-4B chain, Tubulin beta-2B chain, Tubulin beta-2A chain, Tubulin beta-3 chain, Tubulin beta-4A chain, Tubulin beta-6 chain, Tubulin beta-1 chain	FVEWIPNNV	A*02:06	58.7	0.365	P07437 P68371 Q9BVA1 Q13885 Q13509 P04350 Q9BUF5 Q9H4B7	
10	Heat shock protein HSP 90-alpha, Heat shock protein HSP 90-beta, Heat shock protein HSP 90-alpha A2, Putative heat shock protein HSP 90-beta 2	FQAEIAQLMSL	A*02:06	12.9	-0.087	P07900 P08238 Q14568 Q58FF8	
11	Heat shock protein HSP 90-alpha, Heat shock protein HSP 90-beta, Heat shock protein HSP 90-alpha A2, Putative heat shock protein HSP 90-beta 2	FQAEIAQLM	A*02:06	7.2	0.176	P07900 P08238 Q14568 Q58FF8	†
			C*07:02	276.0	0.176		
12	U2 small nuclear ribonucleoprotein B'', U1 small nuclear ribonucleoprotein A	NQFPGFKEV	A*02:06	15.1	0.047	P08579 P09012	
13	Heat shock 70 kDa protein 1A, Heat shock 70 kDa protein 1B, Heat shock 70 kDa protein 1-like	LTIDDGIFEV	A*02:06	6.7	0.403	P0DMV8 P0DMV9 P34931	
14	Translationally-controlled tumor protein, Putative translationally-controlled tumor protein-like protein TPT1P8	EMFSDIYKI	A*02:06	184.5	-0.111	P13693 Q9HAU6	
15	Plastin-3, Plastin-2	ALPEDLVEV	A*02:06	5.1	0.202	P13797 P13796	

16	Receptor-type tyrosine-protein phosphatase delta, Receptor-type tyrosine-protein phosphatase S	GVVDIFQTV	A*02:06	5.3	0.200	P23468<br>Q13332	
17	Rab proteins geranylgeranyltransferase component A 1, Rab proteins geranylgeranyltransferase component A 2	SYYGGNWASF	A*24:02	7.5	0.204	P24386<br>P26374	
			C*07:02	169.2	0.204		
18	DNA dC->dU-editing enzyme APOBEC-3A, DNA dC->dU-editing enzyme APOBEC-3B	LQLDPAQIYRV	A*02:06	57.0	0.086	P31941<br>Q9UH17	
19	GMP reductase 1, GMP reductase 2	GYSEHFVEF	A*24:02	32.7	0.282	P36959<br>Q9P2T1	
			C*07:02	113.3	0.282		
20	Lysine-specific demethylase 5C, Lysine-specific demethylase 5D	NYLDQIAKF	A*24:02	33.2	-0.062	P41229<br>Q9BY66	†
			C*07:02	232.4	-0.062		
21	Small ribosomal subunit protein eS27, Ribosomal protein eS27-like	TVFSHAQTV	A*02:06	53.6	-0.135	P42677<br>Q71UM5	
22	Ribose-phosphate pyrophosphokinase 1, Ribose-phosphate pyrophosphokinase 2, Ribose-phosphate pyrophosphokinase 3	VYAILTHGI	A*24:02	20.3	0.219	P60891<br>P11908<br>P21108	
23	Ubiquitin-conjugating enzyme E2 D3, Ubiquitin-conjugating enzyme E2 D2	SQWSPALTI	A*02:06	24.8	-0.055	P61077<br>P62837	
24	Ubiquitin-conjugating enzyme E2 D3, Ubiquitin-conjugating enzyme E2 D2, Ubiquitin-conjugating enzyme E2 D4	YPFKPPKVAF	B*07:02	44.9	-0.346	P61077<br>P62837<br>Q9Y2X8	
25	Protein transport protein Sec61 subunit alpha isoform 1, Protein transport protein Sec61 subunit alpha isoform 2	RYIPTAAAF	A*24:02	10.2	0.163	P61619<br>Q9H9S3	
			C*07:02	64.3	0.163		

26	14-3-3 protein gamma, 14-3-3 protein eta	QYESKVFYL	A*24:02	393.7	-0.208	P61981<br>Q04917	
27	Small ribosomal subunit protein eS26, Putative ribosomal protein eS26-like	APRPPPKPM	B*07:02	5.2	-0.204	P62854<br>Q5JNZ5	
28	Elongation factor 1-alpha 1, Putative elongation factor 1-alpha-like 3, Elongation factor 1-alpha 2	KYAWVLDKL	A*24:02	34.8	0.158	P68104<br>Q5VTE0<br>Q05639	‡
29	Elongation factor 1-alpha 1, Putative elongation factor 1-alpha-like 3, Elongation factor 1-alpha 2	RPTDKPLRL	B*07:02	68.9	-0.165	P68104<br>Q5VTE0<br>Q05639	
30	Inositol 1, 4, 5-trisphosphate receptor type 2, Inositol 1, 4, 5-trisphosphate receptor type 3, Inositol 1, 4, 5-trisphosphate receptor type 1	IFGVIIDTF	A*24:02	47.4	0.349	Q14571<br>Q14573<br>Q14643	
31	Septin-2, Septin-5, Septin-4	FISPFGHGL	A*02:06	13.9	0.128	Q15019<br>Q99719<br>O43236	
32	Putative Dol-P-Glc:Glc(2)Man(9)GlcNAc(2)-PP-Dol alpha-1,2-glucosyltransferase, Dol-P-Glc:Glc(2)Man(9)GlcNAc(2)-PP-Dol alpha-1,2-glucosyltransferase	LLFSAFSRA	A*02:06	41.3	-0.090	Q5I7T1<br>Q5BKT4	
33	tRNA N(3)-methylcytidine methyltransferase METTL2B, tRNA N(3)-methylcytidine methyltransferase METTL2A	FFKDRHWLF	C*07:02	58.6	0.214	Q6P1Q9<br>Q96IZ6	
			A*24:02	63.9	0.214		
34	Ribonucleoside-diphosphate reductase subunit M2 B, Ribonucleoside-diphosphate reductase subunit M2	KPSEERVREI	B*07:02	188.8	0.336	Q7LG56<br>P31350	
35	Putative ribosomal protein uL10-like, Large ribosomal subunit protein uL10	FLADPSAFVAA	A*02:06	37.2	0.069	Q8NHW5<br>P05388	
36	Folliculin-interacting protein 1, Folliculin-interacting protein 2	PYNPLWAQL	A*24:02	104.5	0.150	Q8TF40<br>Q9P278	

37	SEC14-like protein 1, SEC14-like protein 5	NYPETLGRLLI	A*24:02	371.3	0.190	Q92503<br>O43304	
38	MORF4 family-associated protein 1-like 1, MORF4 family-associated protein 1	FLLPVINEM	A*02:06	6.3	0.204	Q96HT8<br>Q9Y605	
			C*07:02	397.2	0.204		
39	Metastasis-associated protein MTA3, Metastasis-associated protein MTA2	RQIDQFLVV	A*02:06	4.0	0.078	Q9BTC8<br>O94776	
40	Putative FERM domain-containing protein FRMD8P1, FERM domain-containing protein 8	RAADVLVYL	A*02:06	13.4	0.097	Q9BZ68<br>Q9BZ67	‡
41	Ribosomal protein S6 kinase alpha-6, Ribosomal protein S6 kinase alpha-2, Ribosomal protein S6 kinase alpha-1, Ribosomal protein S6 kinase alpha-3	FQTEGKLYL	A*02:06	11.0	-0.068	Q9UK32<br>Q15349<br>Q15418<br>P51812	
			C*07:02	458.5	-0.068		
42	Guanine nucleotide-binding protein-like 3-like protein, Thioredoxin-like protein 4B	VYLDKFIRL	A*24:02	42.9	0.061	Q9NVN8<br>Q9NX01	†
			C*07:02	197.5	0.062		

[#] immunogenicity scores were calculated via the IEDB immunogenicity tool and allele-specific anchor residues were masked. Positive scores indicate more immunogenic peptides, whereas negative scores designate less immunogenic peptides

† peptide sequence identified in two of three mass spectrometry replicates

‡ peptide sequence identified in three of three mass spectrometry replicates
