# Supplementary material for: Gemcitabine Modulates HLA-I Regulation to Improve Tumor Antigen Presentation by Pancreatic Cancer Cells
Source: Int J Mol Sci. 2024 Mar 11;25(6):3211. doi: 10.3390/ijms25063211 (PMC10970070; doi:10.3390/ijms25063211)
Supplement: Supplementary file 1 [file ijms-25-03211-s001.zip › Table S4.pdf]

Table S4. Gene Ontology analysis of source proteins which generated no treatment-exclusive HLA-I peptides.

Ontology domain	*GO term ID*	*Term*	*Count*	*P-value*
MF	GO:0045296	cadherin binding	14	$1.84x10^{-5}$
MF	GO:0000030	mannosyltransferase activity	5	$2.14x10^{-4}$
MF	GO:0003746	translation elongation factor activity	5	$3.28x10^{-4}$
MF	GO:0098973	structural constituent of postsynaptic actin cytoskeleton	3	$5.64x10^{-4}$
MF	GO:0038132	neuregulin binding	3	$1.40x10^{-3}$
MF	GO:0044877	protein-containing complex binding	23	$5.37x10^{-3}$
MF	GO:0140142	nucleocytoplasmic carrier activity	4	$2.14x10^{-2}$
MF	GO:0097718	disordered domain specific binding	4	$3.15x10^{-2}$
MF	GO:0043022	ribosome binding	6	$3.85x10^{-2}$
BP	GO:1901566	organonitrogen compound biosynthetic process	35	$2.42x10^{-7}$
BP	GO:0045901	positive regulation of translational elongation	3	$1.39x10^{-3}$
BP	GO:0045905	positive regulation of translational termination	3	$1.39x10^{-3}$
BP	GO:0097502	mannosylation	5	$3.26x10^{-3}$
BP	GO:1901137	carbohydrate derivative biosynthetic process	16	$3.29x10^{-3}$
BP	GO:0051128	regulation of cellular component organization	33	$7.61x10^{-3}$
BP	GO:0051131	chaperone-mediated protein complex assembly	4	$1.64x10^{-2}$
CC	GO:0070062	extracellular exosome	44	$4.67x10^{-12}$
CC	GO:0005829	cytosol	70	$1.54x10^{-10}$
CC	GO:1904813	ficolin-1-rich granule lumen	9	$1.42x10^{-5}$
CC	GO:0032991	protein-containing complex	74	$1.79x10^{-4}$
CC	GO:0005925	focal adhesion	12	$2.24x10^{-3}$
CC	GO:0012505	endomembrane system	48	$1.08x10^{-2}$
CC	GO:0015629	actin cytoskeleton	12	$1.35x10^{-2}$
CC	GO:0030132	clathrin coat of coated pit	3	$3.07x10^{-2}$

MF, Molecular Function; BP, Biological Process; Cellular Component, CC
