# Supplementary material for: Gemcitabine Modulates HLA-I Regulation to Improve Tumor Antigen Presentation by Pancreatic Cancer Cells
Source: Int J Mol Sci. 2024 Mar 11;25(6):3211. doi: 10.3390/ijms25063211 (PMC10970070; doi:10.3390/ijms25063211)
Supplement: Supplementary file 1 [file ijms-25-03211-s001.zip › Table S5.pdf]

Table S5. Gene Ontology analysis of source proteins which generated gemcitabine-exclusive HLA-I peptides.

Ontology domain	*GO term ID*	*Term*	*Count*	*P-value*
MF	GO:0005515	protein binding	377	$1.44x10^{-18}$
MF	GO:0017111	ribonucleoside triphosphate phosphatase activity	51	$2.05x10^{-12}$
MF	GO:0003723	RNA binding	107	$7.39x10^{-6}$
MF	GO:0008554	P-type sodium transporter activity	5	$4.07x10^{-5}$
MF	GO:0003697	single-stranded DNA binding	14	$1.53x10^{-4}$
MF	GO:0140640	catalytic activity, acting on a nucleic acid	33	$2.75x10^{-4}$
MF	GO:0045182	translation regulator activity	15	$4.57x10^{-4}$
MF	GO:0004711	ribosomal protein S6 kinase activity	4	$3.32x10^{-3}$
MF	GO:0004748	ribonucleoside-diphosphate reductase activity, thioredoxin disulfide as acceptor	3	$4.95x10^{-3}$
MF	GO:0005220	inositol 1,4,5-trisphosphate-sensitive calcium-release channel activity	3	$4.95x10^{-3}$
MF	GO:0000287	magnesium ion binding	16	$1.04x10^{-2}$
MF	GO:0004634	phosphopyruvate hydratase activity	3	$1.95x10^{-2}$
MF	GO:0097718	disordered domain specific binding	6	$2.80x10^{-2}$
MF	GO:0008199	ferric iron binding	4	$2.94x10^{-2}$
MF	GO:0005200	structural constituent of cytoskeleton	10	$4.53x10^{-2}$
MF	GO:0004749	ribose phosphate diphosphokinase activity	3	$4.80x10^{-2}$
MF	GO:0097100	supercoiled DNA binding	3	$4.80x10^{-2}$
BP	GO:0006996	organelle organization	144	$2.72x10^{-17}$
BP	GO:0006518	peptide metabolic process	59	$7.36x10^{-14}$
BP	GO:0051641	cellular localization	121	$7.32x10^{-8}$
BP	GO:0035556	intracellular signal transduction	86	$5.63x10^{-4}$
BP	GO:0010248	establishment or maintenance of transmembrane electrochemical gradient	5	$1.99x10^{-3}$
BP	GO:0006015	5-phosphoribose 1-diphosphate biosynthetic process	4	$3.70x10^{-3}$
BP	GO:0031396	regulation of protein ubiquitination	16	$5.70x10^{-3}$
BP	GO:0051301	cell division	31	$6.46x10^{-3}$
BP	GO:0032502	developmental process	164	$7.70x10^{-3}$
BP	GO:0009056	catabolic process	80	$1.01x10^{-2}$
BP	GO:0030007	intracellular potassium ion homeostasis	5	$1.21x10^{-2}$
BP	GO:0006901	vesicle coating	7	$1.48x10^{-2}$
BP	GO:0036376	sodium ion export across plasma membrane	5	$1.74x10^{-2}$
BP	GO:0034660	ncRNA metabolic process	29	$2.11x10^{-2}$
BP	GO:0010033	response to organic substance	83	$2.95x10^{-2}$
CC	GO:0005737	cytoplasm	338	$1.70x10^{-28}$

CC	GO:0045171	intercellular bridge	15	4.58×10^{-8}
CC	GO:0030054	cell junction	84	5.35×10^{-8}
CC	GO:0098687	chromosomal region	24	1.38×10^{-4}
CC	GO:0000793	condensed chromosome	19	3.63×10^{-4}
CC	GO:0030867	rough endoplasmic reticulum membrane	6	2.68×10^{-3}
CC	GO:0090533	cation-transporting ATPase complex	5	6.47×10^{-3}
CC	GO:0002189	ribose phosphate diphosphokinase complex	3	1.99×10^{-2}
CC	GO:0005784	Sec61 translocon complex	3	3.93×10^{-2}

MF, Molecular Function; BP, Biological Process; Cellular Component, CC
