# Supplementary material for: Gemcitabine Modulates HLA-I Regulation to Improve Tumor Antigen Presentation by Pancreatic Cancer Cells
Source: Int J Mol Sci. 2024 Mar 11;25(6):3211. doi: 10.3390/ijms25063211 (PMC10970070; doi:10.3390/ijms25063211)
Supplement: Supplementary file 1 [file ijms-25-03211-s001.zip › Table S6.pdf]

Table S6. HLA typing of pancreatic cancer cell lines.

Cell line	*Expressed HLA-I molecules*	*Typing source*
S2-013	HLA-A*02:06<br>HLA-A*24:02<br>HLA-B*07:02<br>HLA-B*59:01<br>HLA-C*01:02<br>HLA-C*07:02	Molecular Diagnostics Histocompatibility Lab[#]
T3M-4	HLA-A*02<br>HLA-A*02<br>HLA-B*40<br>HLA-B*52<br>HLA-C*03<br>HLA-C*12	Molecular Diagnostics Histocompatibility Lab[#]
PANC-1	HLA-A*02:01<br>HLA-A*11:01<br>HLA-B*38:01<br>HLA-C*12:03	Tron Cell Line Portal[†]

[#] Molecular Diagnostics Histocompatibility Lab at the University of Nebraska Medical center performed Next Generation Sequencing using the CareDx® AlloSeq™ Tx 17 assay (Illumina MiSeq instruments) on indicated cell types. Sequencing data was compared to the IMGT/HLA database to determine the HLA type for each sample. High resolution and intermediate typing were conducted for the S2-013 and T3M-4 cells, respectively.

[†] Tron Cell Line Portal "http://celllines.tron-mainz.de/ (accessed 10 December 2021)"
